# An alternating-intervention pilot trial on the impact of an informational handout on patient-reported outcomes and follow-up after lung cancer screening

**DOI:** 10.1371/journal.pone.0300352

**Published:** 2024-04-10

**Authors:** Matthew Triplette, Erin K. Kross, Madison Snidarich, Shahida Shahrir, Daniel S. Hippe, Kristina Crothers

**Affiliations:** 1 Public Health Sciences Division, Fred Hutchinson Cancer Center, Seattle, WA, United States of America; 2 Department of Medicine, University of Washington, Seattle, WA, United States of America; 3 Cambia Palliative Care Center of Excellence at UW Medicine, Seattle, WA, United States of America; 4 Veterans Affairs Puget Sound Health Care System, Seattle, WA, United States of America; Vanderbilt University Medical Center, UNITED STATES

## Abstract

**Introduction:**

Lung cancer screening (LCS) can reduce lung cancer mortality; however, poor understanding of results may impact patient experience and follow-up. We sought to determine whether an informational handout accompanying LCS results can improve patient-reported outcomes and adherence to follow-up.

**Study design:**

This was a prospective alternating intervention pilot trial of a handout to accompany LCS results delivery.

**Setting/Participants:**

Patients undergoing LCS in a multisite program over a 6-month period received a mailing containing either: 1) a standardized form letter of LCS results (control) or 2) the LCS results letter and the handout (intervention).

**Intervention:**

A two-sided informational handout on commonly asked questions after LCS created through iterative mixed-methods evaluation with both LCS patients and providers.

**Outcome measures:**

The primary outcomes of 1)patient understanding of LCS results, 2)correct identification of next steps in screening, and 3)patient distress were measured through survey. Adherence to recommended follow-up after LCS was determined through chart review. Outcomes were compared between the intervention and control group using generalized estimating equations.

**Results:**

389 patients were eligible and enrolled with survey responses from 230 participants (59% response rate). We found no differences in understanding of results, identification of next steps in follow-up or distress but did find higher levels of knowledge and understanding on questions assessing individual components of LCS in the intervention group. Follow-up adherence was overall similar between the two arms, though was higher in the intervention group among those with positive findings (p = 0.007).

**Conclusions:**

There were no differences in self-reported outcomes between the groups or overall follow-up adherence. Those receiving the intervention did report greater understanding and knowledge of key LCS components, and those with positive results had a higher rate of follow-up. This may represent a feasible component of a multi-level intervention to address knowledge and follow-up for LCS.

**Trial registration:**

ClinicalTrials.gov NCT05265897.

## Introduction

Lung cancer remains the leading cause of cancer death in the United States. Among high-risk patients, lung cancer screening (LCS) with low-dose chest CT (LDCT) has been recommended by the United States Preventive Services Task Force (USPSTF) since 2013, with eligibility expanded in 2021 [1]. Despite strong evidence, implementation in real-world settings has been poor, with low uptake and suboptimal adherence to annual LDCT and follow-up for those with high-risk findings [2–8]. A recent meta-analysis of LCS adherence showed that the pooled adherence to follow-up in clinical programs is quite low (37%) and our own work demonstrates that nearly half of all patients with positive findings have delayed follow-up [2, 8].

While there are several barriers to effective screening implementation, inadequate patient results understanding may contribute to low adherence to follow-up. High quality shared decision making (SDM) before LCS has the potential to improve patient knowledge [9–11]; however studies suggests SDM is likely underperformed in the clinical setting [12, 13]. Moreover, there are limited tools to facilitate understanding of LCS results *after screening* that have been studied to assess if they improve LCS knowledge, understanding of follow-up, and/or allay distress.

In prior work, we developed a “Common Questions after your Lung Cancer Screening,” (CAQ) handout incorporating patient and provider feedback to improve understanding of screening results [14]. In the current study, we performed a pragmatic alternating-intervention pilot trial of this handout in patients undergoing LCS. While a primary purpose of the pilot was to assess the feasibility and acceptability of the intervention, we also evaluated the impact of the intervention on patient-reported knowledge, understanding and distress as well as adherence with follow-up. We hypothesized that the CAQ would improve overall understanding of screening and next steps in follow-up and reduce distress.

## Methods and materials

### Study population

This study was performed in a Seattle, Washington-based multisite program which oversees LCS provided by 4 tertiary care centers, 27 primary care clinics, specialty services, and self-referred patients. Screening is largely decentralized and managed by primary care providers. Central oversite is used to assist with scheduling and to standardize and track reporting [15]. Screening results are sent to patients via standardized letter by the LCS program within 1 week of screening, with separate letter templates for each Lung-RADS category with additional information for those with “Other significant” or “S-findings.” Other results communication is at the discretion of the ordering physician and may include phone calls, visits, or communication through the electronic health record (EHR). Patients may also access their own LDCT reports through an EHR portal.

### Study design

This was a prospective, alternating-intervention controlled trial of the CAQ handout to accompany screening results [16, 17]. The approved study protocol is included as S1 File. The CAQ handout was based partially on the American Thoracic Society “What is a lung nodule?” handout [18], modified and tailored based on patient focus groups and provider interviews [14]. During the trial, post-screening letter contents alternated on a weekly basis between the standard Lung-RADS based form letter (control) and the form letter with the CAQ handout (intervention). All screening patients undergoing LDCT across the system were included in this allocation scheme from December 7, 2020 through June 14, 2021 (with the exception of a 5-week pause for EHR transition). This included patients undergoing an initial LCS exam (hereafter referred as baseline), or those who had previously had 1 or more LCS exams and were undergoing subsequent annual exams (hereafter referred to as subsequent LDCT) This mailing was sent by LCS clinical staff independent of the study team.

**Fig 1:**
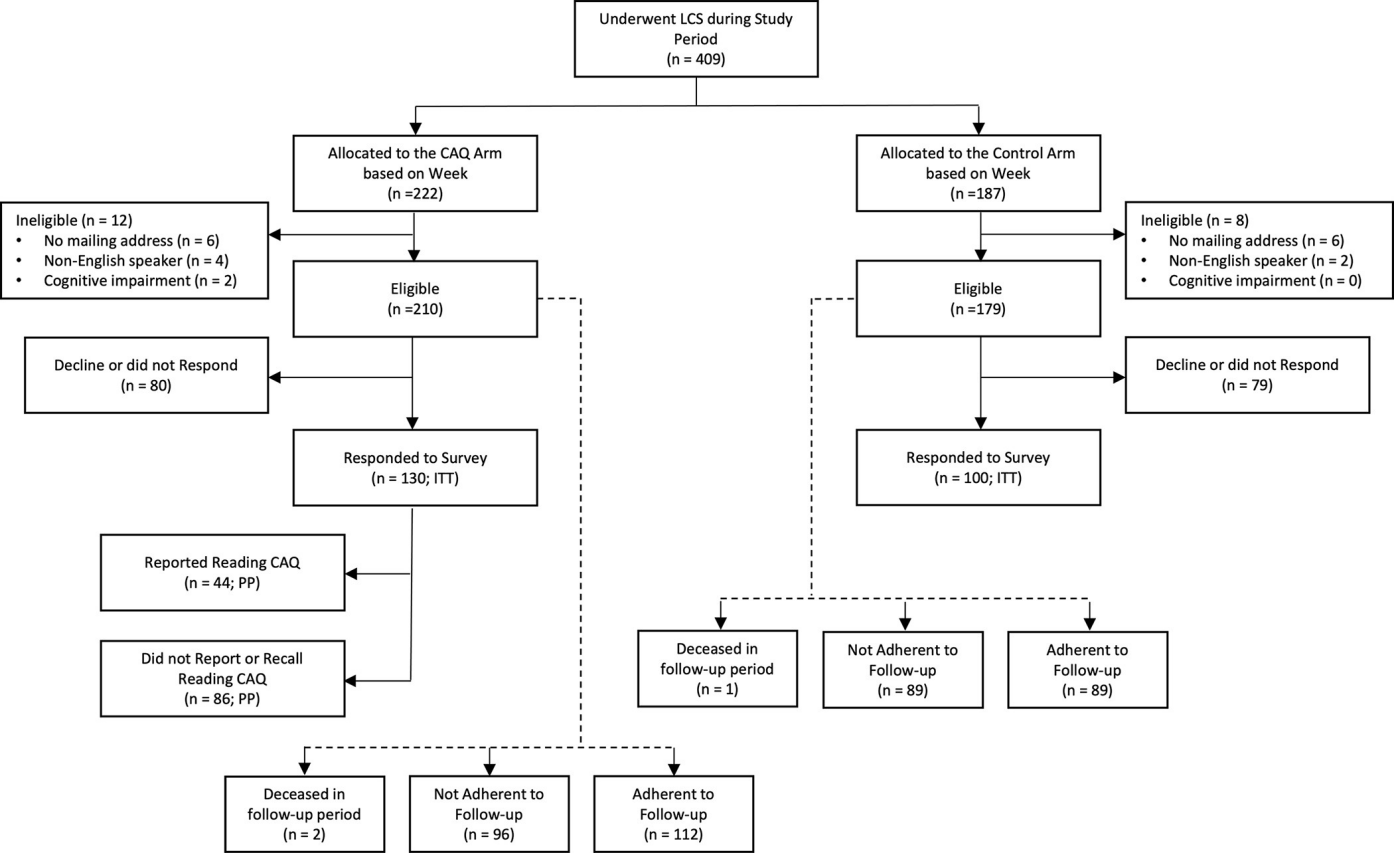
Study flow diagram. Assignment to the CAQ or control arms alternated weekly during the trial period based on the date of screening The participants in the CAQ and control arms that responded to the study survey consistuted the intention-to-treat (ITT) group. The CAQ arm was partitioned into groups of participants who reported reading the CAQ and those who did not report or recall reading the CAQ that were used in a per-protocol (PP) analysis. All participants were followed for adherence to follow-up.

**Fig 2:**
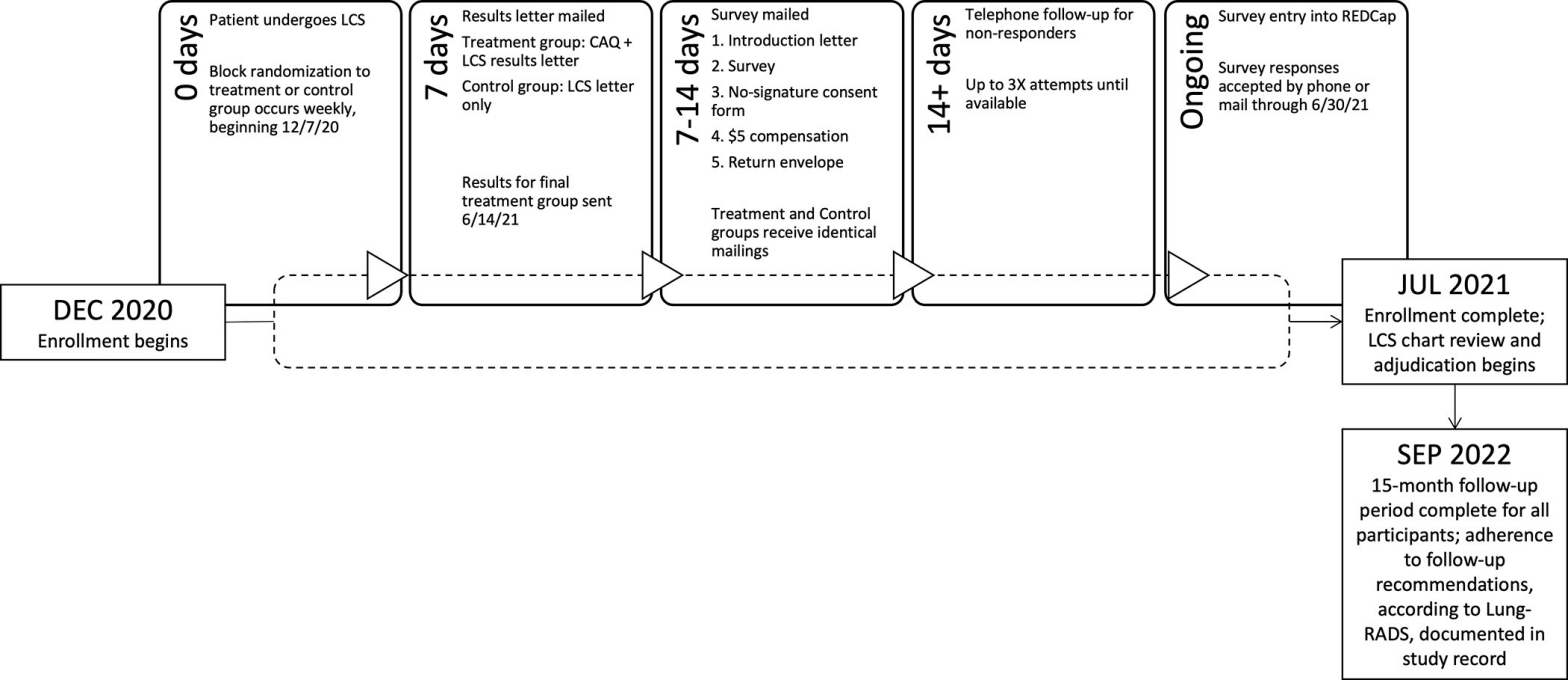
Study enrollment and follow-up timeline. This figure demonstrates the flow through the participation elements from enrollment with randomization, follow-up surveys and adjudication of adherence to follow-up.

One week after sending the results, a second mailing was sent to participants, including an introductory letter, an information statement, a $5 incentive, the survey instrument and a stamped return envelope. After including all participants who underwent a LDCT for lung cancer screening in the alternating intervention-control scheme during the study time period, at this stage, participants who were identified as not capabale of receiving mailings (i.e. returned mailed without a valid alternative mailing address [n = 12]), were identified as non-English speaking documented in the EHR (n = 6) or had cognitive impairment (n = 2) documented in the EHR, were excluded from the study. If surveys were not returned within 2 weeks, a study coordinator called the patient, reminding them to return the survey and offered to complete the survey with them by phone. Up to three reminder phone calls were provided. Study allocation, exclusions and follow-up are shown in Fig 1. The flow and timeline of participation is shown in Fig 2.

This was a partially blinded study with concealment strategies described as follows. Those who sent mailings (intervention) were aware of the assignment but were not involved with the study team or outcome adjudication, LCS ordering providers were not made aware of a participants study assignment or participation in the study and this information was not recorded in the EHR. All data entry during outcome adjudication performed by research staff was done through restricted data entry forms not linked to study assignment. This was partially blinded as patient participants themselves may have been aware of their assignment prior to completing surveys by nature of receiving the mailed intervention.

**Fig 3:**
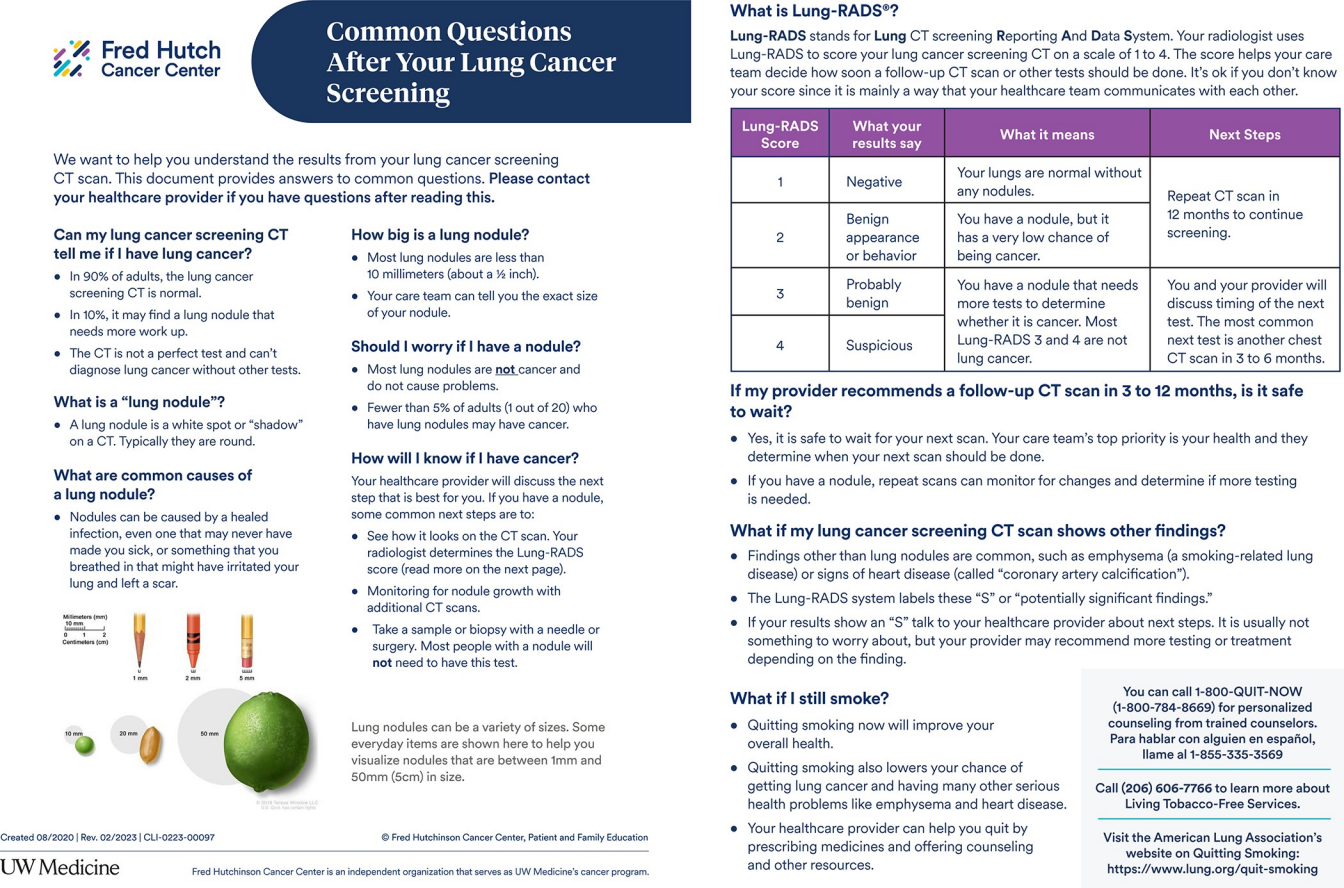
“Common Questions after your Lung Cancer Screening” (CAQ) handout which was mailed to patients in the intervention arm who underwent LCS.

The CAQ handout is a single front-and-back handout (Fig 3). The handout covers topics such as: lung nodules and common causes, next steps after screening, reassurance for waiting for follow-up or annual scans, common incidental findings and smoking cessation information. The handout provides infographics on lung nodule size and the Lung-RADS categorization system. The CAQ handout was created to increase understanding of LCS results, improve adherence to screening follow-up and relieve anxiety around results given the high likelihood of lung nodules and incidental findings on LDCT [14].

### Measures

The primary outcomes were defined by a 94-item survey created by the study team and piloted by two research coordinators. Topical areas of the survey included: demographics and overall health, assessment of understanding of LCS [11], assessment of lung cancer risk, understanding of next screening steps [4], and distress following screening measured by the Impact of Event Scale (IES) [19]. Those who identified on the survey that they received the CAQ completed additional questions to assess the acceptability and appropriateness of the CAQ, using the acceptability of intervention measure (AIM) and intervention appropriateness measure (IAM) [20]. The IES scale is a widely used measure of stress associated with a specific event and has been validated across a range of medical contexts including cancer-related distress [21]. The AIM and IAM demonstrated high construct validity for perception of acceptability and appropriateness of interventions in adopting evidence-based practice in the mental health setting and have since been validated in other context such as postpartum care and physical therapy [22, 23]. The specific measures of understanding of LCS and understanding of next screening steps have associations with measures of health literacy, educational attainment and communication practices of LCS results and are correlated [4]. Survey responses were manually transcribed from paper or telephone conversations into the study database by the research coordinator in an entry form blinded to study assignment.

The 3 primary outcomes derived from the survey were: 1) Response to the question, “I understand the results of my most recent LCS CT scan” on a Likert Scale; 2) Self-report of next step considered correct based on Lung-RADS standardized follow-up (using methods from our prior study) [4]; 3) Distress measured by the IES [19]. Secondary outcomes included: 6 questions assessing knowledge and understanding of components of LCS and summarized measures of acceptability and appropriateness.

We also followed patients longitudinally for at least 15 months (through September 15, 2022) after their enrollment to determine the differences in adherence to recommended LCS follow-up. LCS follow-up was ascertained on all participants who were enrolled in the study, whether or not they returned a survey, through review of the EHR with each participant reviewed by two team members (MT and MS) abstracted into a data entry form and blinded to study assignment. Adherence to follow-up was standardized by Lung-RADS recommendation, consistent with prior study [24]; for patients with Lung-RADS 1 or 2: a chest CT (low-dose or diagnostic) completed within 11–15 months, for Lung-RADS 3: a chest CT completed within 5–8 months, for Lung-RADS 4A: a chest CT completed within 4 months, and for Lung-RADS 4B: a diagnostic image, procedure or provider follow-up consultation within 1 month. Participants who died or had documented move out of area or transferred their care (n = 3) were excluded from this analysis.

### Power calculations

Power calculations were conducted prior to performing the trial. It was expected that 434 patients would undergo LCS during the study period and 160 patients would be eligible and respond to the survey, based on an assumed response rate of 35–40%. We assessed power to detect a difference in one of the primary outcomes: correct identification of next steps of LCS. With 80 participants in each arm, power would range from 76–93% if the rate of incorrect identification of next steps was 45% in the control arm and 20–25% in the CAQ arm, based on a Chi-squared test with two-sided α = 0.05. These estimates were based on a prior study where incorrect identification of next steps was 47% [4].

### Statistical analysis

Fisher’s exact test and Wilcoxon rank-sum tests were used to compare participant characteristics and CT-findings between the intervention and control arms and between the survey responders and non-responders. The primary analyses were based on intention-to-treat group assignment. As part of the secondary analyses, per-protocol groups were defined by partitioning the invention arm into the group who recalled reading the CAQ and the group who did not.

As the primary analysis, we compared differences in outcomes between the CAQ and control arms *a priori* adjusted for LCS indication (baseline vs. subsequent exam) using generalized estimating equation (GEE) based regression models. Comparisons were summarized using the adjusted mean difference for both categorical outcomes and continuous outcomes. Wald tests were used to test differences in binary and continuous outcomes and Wilcoxon rank-sum tests, stratified by LCS indication, were used to test differences in ordinal categorical outcomes and continuous outcomes. Additional sensitivity analyses included: 1) comparing outcomes stratified by LCS indication, 2) repeating the primary analysis but comparing the per-protocol groups, and 3) comparing primary outcomes among those with negative LCS (Lung-RADS 1 and 2). We conducted similar analyses for the secondary survey outcome measures. Among those who recalled reading the CAQ, we calculated mean values with standard deviations of the AIM and IAM score. Finally, we compared rates of adherence to follow-up between the CAQ and control arms and other groups (survey responder vs. non-responder, baseline vs. subsequent exam, and Lung-RADS 1–2 vs. 3-4X) using GEE-based multivariable regression models. Differences between groups were adjusted for the other three group variables. As a sensitivity analysis to ensure roughly equivalent lung cancer risk between arms and analytic subgroups, we also calculated the PLCOm2012 model using survey and screening registry data to estimate a 6-year risk of lung cancer [25]. Detailed regression model output, including all regression coefficients and model fit statistics, are provided in S2 File. All statistical analyses were performed using R (version 4.0.0, R Foundation for Statistical Computing, Vienna Austria). Two-sided p-values <0.05 were considered statistically significant, with no correction for multiple outcomes as this was a pilot trial. Subjects with missing values among the relevant variables were excluded from each analysis separately (complete-case analysis). All outcome analyses were pre-specified and conducted independently using separate models.

### Regulatory information and data sharing

This study was approved by the Fred Hutchinson Cancer Center Institutional Review Board with a waiver of informed consent given minimal risk for both survey participants and non-participants for the minimal EHR review performed in the study. Limited EHR review included protected health information such as medical record number and date of birth. All protected health information was maintained in a password protected study database accessible only to the study team. The institutional review board did not require written signed consent from survey respondents but participants received a study information sheet outlining the study objectives, minimal risks, and returned their surveys as assent for participation.

Institutional Review Board approval for study components is through Fred Hutchinson Cancer Center IRB#9968 and IRB#10082. The study was registered at ClinicalTrials.gov on March 4, 2022, after trial enrollment. Late registration was an oversight on the part of the study team, but registration was complete prior to data analysis. The primary outcomes were established prior to any patient enrollment as outlined in the protocol, finalized October 2020 (S1 File). Data was de-identified with removal of all personal health information and is publically available in the Dryad data repository.

## Results

During the study period 409 patients completed an LCS exam, 389 of which were eligible for the study. Two-hundred-thirty (59%) responded to the survey. Those who responded to the survey were more likely to be undergoing follow-up exams (63% vs. 50%, p = 0.012), have private health insurance (35% vs. 27%, p = 0.026) and have quit smoking (55% vs. 38%, p = 0.001) compared to non-responders (Table 1). Of the 230 study participants, 130 were assigned to the CAQ arm and 100 to the control arm. Of the 130 in the CAQ arm, 44 reported reading the CAQ and 86 did not recall reading the CAQ, defining per-protocol groups. The majority of participants who completed surveys were male (67%) and white (81%) with an average age of 66 (Table 2). Most exams for which participants were receiving results demonstrated normal (Lung-RADS 1 or 2) findings (91%). Study participants between arms were similar and had similar 6-year risks of lung cancer, though PLCOm2012 risk scores could not be calculated for all participants (S3 File).

**Table 1:** Comparison of responders and non-responders (n = 389 eligible patients).

	Participation Status*		
	Responded	Did not Respond	
Variable	(n = 230)	(n = 159)	P-value†
Group assignment			0.26
CAQ	130 (56.5%)	80 (50.3%)	
Control	100 (43.5%)	79 (49.7%)	
Baseline LCS exam (vs. subsequent)	84 (36.5%)	79 (49.7%)	0.012
Male gender‡	155 (67.3%)	102 (64.6%)	0.59
Not recorded	0	1	
Age at screening	66 ± 6	65 ± 6	0.28
Race‡			0.41
White	156 (83.9%)	103 (83.1%)	
Black or African American	15 (8.1%)	15 (12.1%)	
Asian	9 (4.8%)	6 (4.8%)	
American Indian or Alaska Native	4 (2.2%)	0 (0%)	
Native Hawaiian or other Pacific Islander	1 (0.5%)	0 (0%)	
Other race	0 (0%)	0 (0%)	
Multiple races	1 (0.5%)	0 (0%)	
Not recorded	44	35	
Ethnicity‡			>0.99
Hispanic or Latino	4 (5.1%)	2 (3.3%)	
Not Hispanic or Latino	74 (94.9%)	58 (96.7%)	
Not recorded	152	99	
Insurance status‡			0.026
Private/HMO	67 (35.3%)	36 (27.1%)	
Medicare + Private	6 (3.2%)	0 (0%)	
Medicare	86 (45.3%)	640 (48.1%)	
Medicaid	30 (15.8%)	29 (21.8%)	
Other	1 (0.5%)	4 (3.0%)	
Not recorded	40	26	
Current smoker‡	104 (45.6%)	98 (62.0%)	0.002
Not recorded	2	1	
Pack-years (if smoked)‡	47 (40–70)	50 (39–64)	0.99
Not recorded	7	5	
Lung-RADS			0.99
1	65 (28.3%)	48 (30.2%)	
2	144 (62.6%)	98 (61.6%)	
3	8 (3.5%)	5 (3.1%)	
4A	9 (3.9%)	6 (3.8%)	
4B	3 (1.3%)	1 (0.6%)	
4X	1 (0.4%)	1 (0.6%)	

*Values are no. (%), mean ± SD or median (inter-quartile range); the number of subjects with missing values are summarized separately under each item with at least one missing value

†Fisher’s exact test or the Wilcoxon rank-sum test comparing the two groups

‡For accurate comparison between survey respondents and non-respondents these measures are taken from universal lung cancer screening registry, which may differ from the values reported on the study survey.

**Table 2:** Survey-respondant characteristics (n = 230).

			Group Assignment*	
	All*	CAQ Arm	Control Arm	
Variable	(n = 230)	(n = 130)	(n = 100)	P-value†
Baseline LCS exam (vs. subsequent)	84 (36.5%)	53 (40.8%)	31 (31.0%)	0.13
Male gender	155 (67.4%)	90 (69.2%)	65 (65.0%)	0.57
Age at screening	66 ± 6	66 ± 7	66 ± 6	0.51
Race				0.87
White	187 (81.3%)	106 (81.5%)	81 (81.0%)	
Black or African American	15 (6.5%)	9 (6.9%)	6 (6.0%)	
Asian	12 (5.2%)	6 (4.6%)	6 (6.0%)	
American Indian or Alaska Native	5 (2.2%)	2 (1.5%)	3 (3.0%)	
Native Hawaiian or other Pacific Islander	3 (1.3%)	1 (0.8%)	2 (2.0%)	
Other race	1 (0.4%)	1 (0.8%)	0 (0%)	
Multiple races	7 (3.0%)	5 (3.8%)	2 (2.0%)	
Ethnicity				0.17
Hispanic or Latino	5 (2.2%)	1 (0.8%)	4 (4.1%)	
Not Hispanic or Latino	220 (97.8%)	126 (99.2%)	94 (95.9%)	
Did not respond	5	3	2	
Employment status				0.48
Employed/student	60 (26.2%)	31 (23.8%)	29 (29.3%)	
Retired	123 (53.7%)	74 (56.9%)	49 (49.5%)	
Disabled	28 (12.2%)	17 (13.1%)	11 (11.1%)	
Unemployed/other	18 (7.9%)	8 (6.2%)	10 (10.1%)	
Did not respond	1	0	1	
Education				0.48
High school or less	67 (29.4%)	41 (31.8%)	26 (26.3%)	
Some college	72 (31.6%)	42 (32.6%)	30 (30.3%)	
College graduate or more	89 (39.0%)	46 (35.7%)	43 (43.4%)	
Did not respond	2	1	1	
Insurance status				0.067
Private/HMO	64 (28.1%)	39 (30.2%)	25 (25.3%)	
Medicare + Private	37 (16.2%)	21 (16.3%)	16 (16.2%)	
Medicare	72 (31.6%)	35 (27.1%)	37 (37.4%)	
Medicaid	17 (7.5%)	8 (6.2%)	9 (9.1%)	
Medicaid + Medicare	23 (10.1%)	19 (14.7%)	4 (4.0%)	
Other	15 (6.6%)	7 (5.4%)	8 (8.1%)	
Did not respond	2	1	1	
Current smoker	109 (47.4%)	62 (47.7%)	47 (47.0%)	>0.99
Pack-years	38 (25–47)	36 (26–50)	38 (25–47)	0.72
Lung-RADS				0.11
1	65 (28.3%)	32 (24.6%)	33 (33.0%)	
2	144 (62.6%)	82 (63.1%)	62 (62.0%)	
3	8 (3.5%)	4 (3.1%)	4 (4.0%)	
4A	9 (3.9%)	8 (6.2%)	1 (1.0%)	
4B	3 (1.3%)	3 (2.3%)	0 (0%)	
4X	1 (0.4%)	1 (0.8%)	0 (0%)	
Significant other (S) findings present	49 (21.3%)	29 (22.3%)	20 (20.0%)	0.67

*Values are no. (%), mean ± SD or median (inter-quartile range); the number of subjects with missing values (item-level non-response) are summarized separately under each item with at least one missing value

†Fisher’s exact test or the Wilcoxon rank-sum test comparing the two groups.

In the total cohort, 58% “strongly agreed” that they understood their most recent LCS CT scan, 82% correctly identified their next step in screening, and IES scores reflected low levels of distress (mean: 11; median: 5) (Table 3). In the primary analyses, there were no statistically significant differences in the primary outcomes of understanding of results (57% vs. 58%, p = 0.93), correctness of self-reported next step (84% vs. 80%, p = 0.35) and distress (median: 6 vs. 3, p = 0.19).

**Table 3:** Comparison of the primary study outcomes between intention-to-treat groups (n = 230).

		Group Assignment*		Adjusted Difference†		
	All*	CAQ Arm	Control Arm	(CAQ–Control)		
Primary Outcome	(n = 230)	(n = 130)	(n = 100)	Mean	(95% CI)	P-value
1) “I understand the results of my most recent LCS CT scan”						0.93‡
Strongly agree	130 (57.5%)	73 (57.0%)	57 (58.2%)	-0.8%	(-13.9, 12.3%)	
Somewhat agree	66 (29.2%)	38 (29.7%)	28 (28.6%)	1.0%	(-11.2, 13.1%)	
Somewhat disagree	22 (9.7%)	14 (10.9%)	8 (8.2%)	2.6%	(-5.1, 10.4%)	
Strongly disagree	8 (3.5%)	3 (2.3%)	5 (5.1%)	-2.8%	(-8.0, 2.4%)	
Did not respond	4	2	2			
2) Self-reported next step is correct	185 (81.9%)	107 (83.6%)	78 (79.6%)	4.9%	(-5.4, 15.2%)	0.35
Did not respond	4	2	2			
3) Impact of Event Scale (IES)§						
Mean ± SD	11 ± 14	11 ± 14	10 ± 14	1.2	(-2.4, 4.9)	0.51
Median (IQR)	5 (0–16)	6 (0–18)	3 (0–14)			0.19‡
Did not respond	3	0	3			

*Values are no. (%), mean ± SD, or median (IQR); the number of subjects with missing values (item-level non-response) are summarized separately under each item with at least one missing values

†Primary analysis, with differences adjusted for LCS indication (baseline vs. subsequent LCS exam)

‡Wilcoxon rank-sum test stratified on LCS indication

§IES can range from 0 to 75; higher values indicate more distress.

There were differences in 4 of 6 secondary outcomes about LCS knowledge (Table 4). In particular, the CAQ group was more likely to strongly or somewhat agree with statements “I feel informed about what a lung nodule is” (72% vs. 49%, p = 0.005), “I am confident that I could tell a family member or friend what a lung nodule is” (62% vs. 41%, p = 0.005), “Most lung nodules are not cancer” (83% vs. 73%, p = 0.031), “I feel informed about what a Lung-RADS score means” (31% vs. 16%, p < 0.001).

**Table 4:** Comparison of the secondary study outcomes between intention-to-treat groups (n = 230).

		Group Assignment*		Adjusted Difference†		
	All*	CAQ Arm	Control Arm	(CAQ–Control)		
Secondary Outcome	(n = 230)	(n = 130)	(n = 100)	Mean	(95% CI)	P-value
“I feel informed about what a lung nodule is”						0.005
Strongly agree	67 (30.0%)	42 (33.3%)	25 (25.8%)	7.3%	(-4.6, 19.1%)	
Somewhat agree	72 (32.3%)	49 (38.9%)	23 (23.7%)	16.0%	(3.9, 28.0%)	
Somewhat disagree	51 (22.9%)	22 (17.5%)	29 (29.9%)	-12.9%	(-24.2, -1.6%)	
Strongly disagree	33 (14.8%)	13 (10.3%)	20 (20.6%)	-10.3%	(-20.4, -0.3%)	
Did not respond	7	4	3			
“I am confident that I could tell a family member or friend what a lung nodule is”						0.005
Strongly agree	49 (21.6%)	28 (21.9%)	21 (21.2%)	0.8%	(-9.7, 11.4%)	
Somewhat agree	72 (31.7%)	52 (40.6%)	20 (20.2%)	21.0%	(9.5, 32.6%)	
Somewhat disagree	47 (20.7%)	26 (20.3%)	21 (21.2%)	-1.6%	(-12.3, 9.1%)	
Strongly disagree	59 (26.0%)	22 (17.2%)	37 (37.4%)	-20.3%	(-32.1, -8.5%)	
Did not respond	3	2	1			
“Most lung nodules are not cancer”						0.031
Strongly agree	63 (30.4%)	42 (35.3%)	21 (23.9%)	10.8%	(-1.6, 23.2%)	
Somewhat agree	100 (48.3%)	57 (47.9%)	43 (48.9%)	-0.4%	(-14.3, 13.6%)	
Somewhat disagree	31 (15.0%)	14 (11.8%)	17 (19.3%)	-7.6%	(-18.1, 2.9%)	
Strongly disagree	13 (6.3%)	6 (5.0%)	7 (8.0%)	-2.9%	(-10.2, 4.5%)	
Did not respond	23	21	12			
“I feel informed about what a Lung-RADS score means”						<0.001
Strongly agree	19 (8.6%)	13 (10.4%)	6 (6.2%)	4.2%	(-2.9, 11.3%)	
Somewhat agree	36 (16.2%)	26 (20.8%)	10 (10.3%)	10.6%	(1.3, 19.9%)	
Somewhat disagree	58 (26.1%)	39 (31.2%)	19 19.6%)	12.0%	(0.6, 23.3%)	
Strongly disagree	109 (49.1%)	47 (37.6%)	62 (63.9%)	-26.8%	(-39.6, -14.0%)	
Did not respond	8	5	3			
“Lung cancer screening can find things in the chest that are not cancer but I might need an extra test to check”						0.66
Strongly agree	120 (53.3%)	70 (55.1%)	50 (51.0%)	4.9%	(-8.3, 18.1%)	
Somewhat agree	85 (37.8%)	45 (35.4%)	40 (40.8%)	-6.0%	(-18.9, 6.8%)	
Somewhat disagree	9 (4.0%)	5 (3.9%)	4 (4.1%)	-0.3%	(-5.4, 4.8%)	
Strongly disagree	11 (4.9%)	7 (5.5%)	4 (4.1%)	1.4%	(-4.4, 7.2%)	
Did not respond	5	3	2			
“It is safe to wait for the next recommended CT scan even if someone has a lung nodule on their lung cancer screening CT”						0.58
Strongly agree	53 (24.7%)	33 (27.0%)	20 (21.5%)	6.5%	(-4.8, 17.8%)	
Somewhat agree	92 (42.8%)	49 (40.2%)	43 (46.2%)	-6.0%	(-19.5, 7.4%)	
Somewhat disagree	38 (17.7%)	23 (18.9%)	15 (16.1%)	2.2%	(-8.1, 12.5%)	
Strongly disagree	32 (14.9%)	17 (13.9%)	15 (16.1%)	-2.6%	(-12.5, 7.3%)	
Did not respond	15	8	7			

*Values are no. (%); the number of subjects with missing values (item-level non-response) are summarized separately under each item with at least one missing value

†Primary analysis, with differences adjusted for LCS indication (baseline vs. subsequent LCS exam).

**Fig 4:**
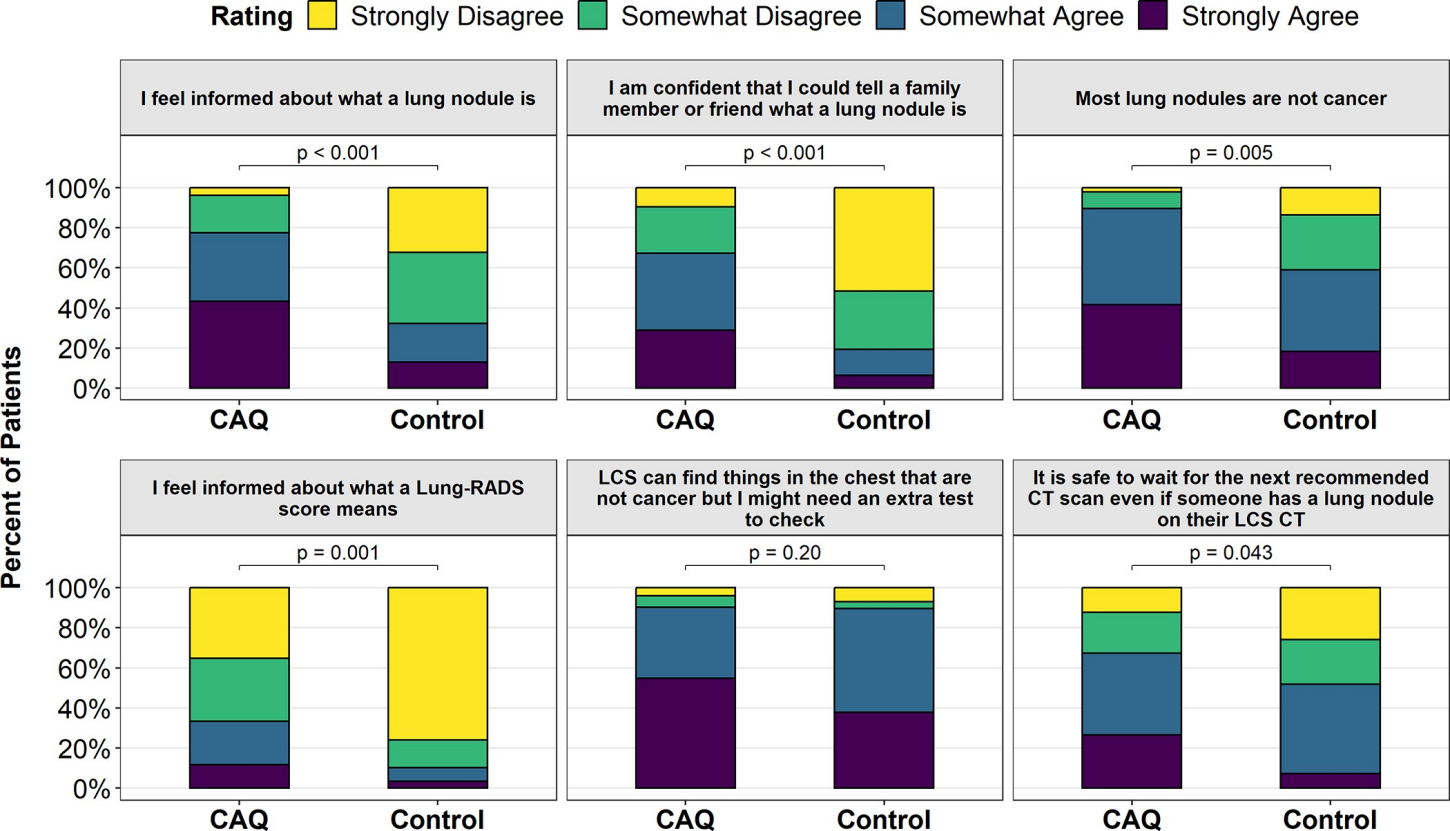
Comparison of answers on 6 understanding and knowledge questions among baseline screening participants between the CAQ arm and control arm.

We found no significant differences in the primary outcomes between treatment arms within either the baseline or subsequent LCS exam strata. However, significantly higher scores in 5 of the 6 measures of LCS knowledge were seen in the CAQ group vs. control group for participants undergoing a baseline scan, while significantly higher measures were only seen in 1 of 6 measures of LCS knowledge for participants undergoing a subsequent exam (Table 5, Fig 4). Using a per-protocol approach there were no significant differences in the primary outcomes between any of the three groups (Table 6). However, among the 4 of 6 secondary LCS knowledge outcomes that were significantly higher in CAQ group in the intention-to-treatment analysis, the group that reported reading the CAQ had higher knowledge scores than both the control group (p<0.004 for each) and the group within the CAQ arm that did not report reading the CAQ (p = 0.030–0.095 for each). There were no differences in primary outcomes when limiting the analytic cohort to those with negative exams.

**Table 5:** Comparison of the primary and secondary study outcomes between intention-to-treat groups, stratified by baseline vs. subsequent LCS exam (n = 230).

	Baseline LCS Exam					Subsequent LCS Exam					Difference of
	Group Assignment*		Difference			Group Assignment*		Difference			Differences
	CAQ Arm	Control Arm	(CAQ—Control)			CAQ Arm	Control Arm	(CAQ—Control)			(Interaction)†
Primary Outcome	(n = 53)	(n = 31)	Mean	(95% CI)	P-value	(n = 77)	(n = 69)	Mean	(95% CI)	P-value	P-value
“I understand the results of my most recent LCS CT scan”					0.12					0.23	0.050
Strongly agree	33 (62.3%)	13 (43.3%)	18.9%	(-3.1, 40.9%)		40 (53.3%)	44 (64.7%)	-11.4%	(-27.4, 4.6%)		
Somewhat agree	13 (24.5%)	12 (40.0%)	-15.5%	(-36.5, 5.5%)		25 (33.3%)	16 (23.5%)	9.8%	(-4.9, 24.5%)		
Somewhat disagree	6 (11.3%)	3 (10.0%)	1.3%	(-12.4, 15.0%)		8 (10.7%)	5 (7.4%)	3.3%	(-6.0, 12.7%)		
Strongly disagree	1 (1.9%)	2 (6.7%)	-4.8%	(-14.4, 4.9%)		2 (2.7%)	3 (4.4%)	-1.7%	(-7.8, 4.3%)		
Did not respond	0	1				2	1				
Self-reported next step is correct	43 (81.1%)	21 (70.0%)	11.1%	(-8.4, 30.6%)	0.25	64 (85.3%)	57 (83.8%)	1.5%	(-10.4, 13.4%)	0.80	0.48
Did not respond	0	1				2	1				
Impact of Event Scale (IES)											
Mean ± SD	15 ± 16	11 ± 15	4.1	(-2.9, 11.0)	0.25	9 ± 13	9 ± 13	-0.2	(-4.4, 4.0)	0.92	0.30
Median (IQR)	10 (2–24)	4 (0–15)			0.14	3 (0–12)	3 (0–13)			0.86	
Did not respond	0	3				0	0				
**Secondary Outcome**											
“I feel informed about what a lung nodule is”					<0.001					0.79	0.001
Strongly agree	23 (43.4%)	4 (12.9%)	30.5%	(12.7, 48.3%)		19 (26.0%)	21 (31.8%)	-5.8%	(-20.9, 9.3%)		
Somewhat agree	18 (34.0%)	6 (19.4%)	14.6%	(-4.3, 33.5%)		31 (42.5%)	17 (25.8%)	16.7%	(1.2, 32.2%)		
Somewhat disagree	10 (18.9%)	11 (35.5%)	-16.6%	(-36.5, 3.2%)		12 (16.4%)	18 (27.3%)	-10.8%	(-24.5, 2.9%)		
Strongly disagree	2 (3.8%)	10 (32.3%)	-28.5%	(-45.7, -11.2%)		11 (15.1%)	10 (15.2%)	-0.1%	(-12.0, 11.8%)		
Did not respond	0	0				4	3				
“I am confident that I could tell a family member or friend what a lung nodule is”					<0.001					0.85	<0.001
Strongly agree	15 (28.8%)	2 (6.5%)	22.4%	(7.3, 37.4%)		13 (17.1%)	19 (27.9%)	-10.8%	(-24.5, 2.8%)		
Somewhat agree	20 (38.5%)	4 (12.9%)	25.6%	(7.8, 43.3%)		32 (42.1%)	16 (23.5%)	18.6%	(3.6, 33.6%)		
Somewhat disagree	12 (23.1%)	9 (29.0%)	-6.0%	(-25.6, 13.7%)		14 (18.4%)	12 (17.6%)	0.8%	(-11.8, 13.3%)		
Strongly disagree	5 (9.6%)	16 (51.6%)	-42.0%	(-61.3, -22.7%)		17 (22.4%)	21 (30.9%)	-8.5%	(-22.9, 5.9%)		
Did not respond	1	0				1	1				
“Most lung nodules are not cancer”					0.005					0.59	0.032
Strongly agree	20 (41.7%)	4 (18.2%)	23.5%	(2.2, 44.8%)		22 (31.0%)	17 (25.9%)	5.2%	(-9.8, 20.3%)		
Somewhat agree	23 (47.9%)	9 (40.9%)	7.0%	(-17.9, 31.9%)		34 (47.9%)	34 (51.5%)	-3.6%	(-20.4, 13.1%)		
Somewhat disagree	4 (8.3%)	6 (27.3%)	-18.9%	(-39.1, 1.2%)		10 (14.1%)	11 (16.7%)	-2.6%	(-14.7, 9.5%)		
Strongly disagree	1 (2.1%)	3 (13.6%)	-11.6%	(-26.5, 3.3%)		5 (7.0%)	4 (6.1%)	1.0%	(-7.3, 9.3%)		
Did not respond	5	9				6	3				
“I feel informed about what a Lung-RADS score means”					0.001					0.028	0.10
Strongly agree	6 (11.8%)	1 (3.4%)	8.3%	(-2.7, 19.4%)		7 (9.5%)	5 (7.4%)	2.1%	(-7.0, 11.2%)		
Somewhat agree	11 (21.6%)	2 (6.9%)	14.7%	(0.1, 29.2%)		15 (20.3%)	8 (11.8%)	8.5%	(-3.4, 20.4%)		
Somewhat disagree	16 (31.4%)	4 (13.8%)	17.6%	(-0.3, 35.5%)		23 (31.1%)	15 (22.1%)	9.0%	(-5.4, 23.5%)		
Strongly disagree	18 (35.3%)	22 (75.9%)	-40.6%	(-60.9, -20.2%)		29 (39.2%)	40 (58.8%)	-19.6%	(-35.8, -3.5%)		
Did not respond	2	2				3	1				
“Lung cancer screening can find things in the chest that are not cancer but I might need an extra test to check”					0.20					0.79	0.23
Strongly agree	28 (54.9%)	11 (37.9%)	17.0%	(-5.4, 39.3%)		42 (55.3%)	39 (56.5%)	-1.3%	(-17.4, 14.9%)		
Somewhat agree	18 (35.3%)	15 (51.7%)	-16.4%	(-38.9, 6.0%)		27 (35.5%)	25 (36.2%)	-0.7%	(-16.3, 14.9%)		
Somewhat disagree	3 (5.9%)	1 (3.4%)	2.4%	(-6.8, 11.7%)		2 (2.6%)	3 (4.3%)	-1.7%	(-7.7, 4.3%)		
Strongly disagree	2 (3.9%)	2 (6.9%)	-3.0%	(-13.6, 7.7%)		5 (6.6%)	2 (2.9%)	3.7%	(-3.2, 10.5%)		
Did not respond	2	2				1	0				
“It is safe to wait for the next recommended CT scan even if someone has a lung nodule on their lung cancer screening CT”					0.043					0.59	0.047
Strongly agree	13 (26.5%)	2 (7.4%)	19.1%	(3.3, 34.9%)		20 (27.4%)	18 (27.3%)	0.1%	(-14.7, 15.0%)		
Somewhat agree	20 (40.8%)	12 (44.4%)	-3.6%	(-26.9, 19.6%)		29 (39.7%)	31 (47.0%)	-7.2%	(-23.7, 9.2%)		
Somewhat disagree	10 (20.4%)	6 (22.2%)	-1.8%	(-21.1, 17.5%)		13 (17.8%)	9 (13.6%)	4.2%	(-7.9, 16.2%)		
Strongly disagree	6 (12.2%)	7 (25.9%)	-13.7%	(-32.6, 5.2%)		11 (15.1%)	8 (12.1%)	2.9%	(-8.4, 14.3%)		
Did not respond	4	4				4	3				

*Values are no. (%), mean ± SD, or median (IQR); the number of subjects with missing values (item-level non-response) are summarized separately under each item with at least one missing value

†Inclusion of interaction terms representing the assignment arm x LCS indication (baseline vs. subsequent LCS exam) to the generalized estimating equation (GEE) models to test for a difference in the differences between the CAQ and control arms within each LCS indication stratum.

**Table 6:** Comparison of the primary and secondary study outcomes between per-protocol groups (n = 230).

	Group Assignment*			Adjusted Difference†			Adjusted Difference‡			Adjusted Difference†		
	CAQ-Read	CAQ-Unread	Control	(CAQ-Read—Control)			(CAQ-Read—CAQ-Unread)			(CAQ-Unread—Control)		
Primary Outcome	(n = 44)	(n = 86)	(n = 100)	Mean	(95% CI)	P-value‡	Mean	(95% CI)	P-value‡	Mean	(95% CI)	P-value‡
“I understand the results of my most recent LCS CT scan”						0.53			0.39			0.78
Strongly agree	23 (53.5%)	50 (58.8%)	57 (58.2%)	-4.3%	(-22.2, 13.6%)		-5.3%	(-23.6, 12.9%)		1.0%	(-13.4, 15.4%)	0.89
Somewhat agree	12 (27.9%)	26 (30.6%)	28 (28.6%)	-0.8%	(-17.1, 15.4%)		-2.7%	(-19.3, 13.9%)		1.9%	(-11.6, 15.4%)	0.79
Somewhat disagree	6 (14.0%)	8 (9.4%)	8 (8.2%)	5.6%	(-6.1, 17.3%)		4.5%	(-7.5, 16.6%)		1.1%	(-7.3, 9.5%)	0.79
Strongly disagree	2 (4.7%)	1 (1.2%)	5 (5.1%)	-0.5%	(-8.4, 7.4%)		3.5%	(-3.3, 10.2%)		-4.0%	(-8.9, 0.9%)	0.16
Did not respond	1	1	2									
Self-reported next step is correct	35 (81.4%)	72 (84.7%)	78 (79.6%)	2.7%	(-11.8, 17.2%)	0.71§	-3.3%	(-17.3, 10.8%)	0.64§	6.0%	(-5.0, 16.9%)	0.29§
Did not respond	1	1	2									
Impact of Event Scale (IES)||												
Mean ± SD	12 ± 16	11 ± 13	10 ± 14	1.8	(-3.6, 7.3)	0.51§	0.9	(-4.4, 6.3)	0.73§	0.9	(-2.9, 4.7)	0.65§
Median (IQR)	6 (0–17)	6 (0–19)	3 (0–14)			0.24			0.86			0.29
Did not respond	0	0	3									
**Secondary Endpoint**												
“I feel informed about what a lung nodule is”						0.004			0.095			0.044
Strongly agree	19 (44.2%)	23 (27.7%)	25 (25.8%)	18.2%	(1.0, 35.3%)		16.5%	(-1.1, 34.1%)		1.7%	(-11.1, 14.5%)	
Somewhat agree	15 (34.9%)	34 (41.0%)	23 (23.7%)	11.9%	(-4.7, 28.5%)		-6.1%	(-23.8, 11.6%)		18.0%	(4.5, 31.6%)	
Somewhat disagree	4 (9.3%)	18 (21.7%)	29 (29.9%)	-21.1%	(-33.7, -8.5%)		-12.4%	(-24.8, 0.0%)		-8.7%	(-21.5, 4.1%)	
Strongly disagree	5 (11.6%)	8 (9.6%)	20 (20.6%)	-9.0%	(-21.8, 3.8%)		2.0%	(-9.5, 13.5%)		-11.0%	(-21.6, -0.4%)	
Did not respond	1	3	3									
“I am confident that I could tell a family member or friend what a lung nodule is”						0.001			0.020			0.080
Strongly agree	14 (32.6%)	14 (16.5%)	21 (21.2%)	11.5%	(-4.6, 27.5%)		16.1%	(-0.1, 32.2%)		-4.6%	(-15.6, 6.4%)	
Somewhat agree	18 (41.9%)	34 (40.0%)	20 (20.2%)	22.2%	(5.5, 38.9%)		1.8%	(-16.3, 19.8%)		20.4%	(7.4, 33.5%)	
Somewhat disagree	6 (14.0%)	20 (23.5%)	21 (21.2%)	-7.9%	(-21.1, 5.4%)		-9.5%	(-23.2, 4.3%)		1.6%	(-10.5, 13.7%)	
Strongly disagree	5 (11.6%)	17 (20.0%)	37 (37.4%)	-25.8%	(-39.5, -12.1%)		-8.4%	(-21.2, 4.5%)		-17.5%	(-30.5, -4.5%)	
Did not respond	1	1	1									
“Most lung nodules are not cancer”						0.004			0.047			0.27
Strongly agree	20 (46.5%)	22 (28.9%)	21 (23.9%)	22.0%	(4.6, 39.4%)		17.5%	(-0.5, 35.5%)		4.5%	(-9.0, 18.1%)	
Somewhat agree	18 (41.9%)	39 (51.3%)	43 (48.9%)	-6.4%	(-24.6, 11.8%)		-9.4%	(-27.9, 9.2%)		3.0%	(-12.5, 18.5%)	
Somewhat disagree	4 (9.3%)	10 (13.2%)	17 (19.3%)	-10.1%	(-22.5, 2.3%)		-3.9%	(-15.4, 7.7%)		-6.2%	(-17.7, 5.3%)	
Strongly disagree	1 (2.3%)	5 (6.6%)	7 (8.0%)	-5.6%	(-13.3, 2.2%)		-4.2%	(-11.4, 2.9%)		-1.3%	(-9.7, 7.0%)	
Did not respond	1	10	12									
“I feel informed about what a Lung-RADS score means”						<0.001			0.030			0.009
Strongly agree	6 (14.6%)	7 (8.3%)	6 (6.2%)	8.5%	(-3.4, 20.3%)		6.3%	(-6.0, 18.6%)		2.2%	(-5.3, 9.6%)	
Somewhat agree	12 (29.3%)	14 (16.7%)	10 (10.3%)	19.1%	(3.9, 34.3%)		12.6%	(-3.4, 28.7%)		6.5%	(-3.5, 16.5%)	
Somewhat disagree	12 (29.2%)	27 (32.1%)	19 (19.6%)	10.0%	(-5.9, 26.0%)		-2.8%	(-20.0, 14.3%)		12.9%	(0.2, 25.6%)	
Strongly disagree	11 (26.8%)	36 (42.9%)	62 (63.9%)	-37.6%	(-54.3, -21.0%)		-16.1%	(-33.3, 1.2%)		-21.5%	(-35.8, -7.3%)	
Did not respond	3	2	3									
“Lung cancer screening can find things in the chest that are not cancer but I might need an extra test to check”						0.38			0.41			0.96
Strongly agree	27 (61.4%)	43 (51.8%)	50 (51.0%)	11.2%	(-6.3, 28.7%)		9.6%	(-8.4, 27.7%)		1.6%	(-13.1, 16.2%)	
Somewhat agree	12 (27.3%)	33 (39.8%)	40 (40.8%)	-14.2%	(-30.7, 2.2%)		-12.6%	(-29.4, 4.3%)		-1.7%	(-16.1, 12.7%)	
Somewhat disagree	4 (9.1%)	1 (1.2%)	4 (4.1%)	4.8%	(-4.5, 14.1%)		7.9%	(-0.9, 16.7%)		-3.0%	(-7.5, 1.4%)	
Strongly disagree	1 (2.3%)	6 (7.2%)	4 (4.1%)	-1.8%	(-8.0, 4.4%)		-5.0%	(-12.1, 2.2%)		3.1%	(-3.8, 10.1%)	
Did not respond	0	3	2									
“It is safe to wait for the next recommended CT scan even if someone has a lung nodule on their lung cancer screening CT”						0.11			0.089			0.80
Strongly agree	13 (31.0%)	20 (25.0%)	20 (21.5%)	10.6%	(-5.4, 26.7%)		6.3%	(-10.5, 23.1%)		4.3%	(-8.1, 16.8%)	
Somewhat agree	19 (45.2%)	30 (37.5%)	43 (46.2%)	-0.9%	(-19.2, 17.3%)		7.8%	(-10.7, 26.2%)		-8.7%	(-23.4, 6.1%)	
Somewhat disagree	9 (21.4%)	14 (17.5%)	15 (16.1%)	4.6%	(-9.7, 18.9%)		3.7%	(-11.0, 18.5%)		0.9%	(-10.5, 12.3%)	
Strongly disagree	1 (2.4%)	16 (20.0%)	15 (16.1%)	-14.3%	(-23.6, -5.1%)		-17.8%	(-27.8, -7.8%)		3.5%	(-8.2, 15.1%)	
Did not respond	2	6	7									

*Values are no. (%), mean ± SD, or median (IQR); the number of subjects with missing values (item-level non-response) are summarized separately under each item with at least one missing value

†Differences between groups adjusted for LCS indication (baseline vs. subsequent LCS exam)

‡Wilcoxon rank-sum test stratified on LCS indication except where indicated

§Wald test from corresponding generalized estimating equations model.

We evaluated the acceptability and appropriateness to deliver information to accompany the screening results among the 44 participants in the CAQ arm who reported reading the information sheet. On scales of 1 (completely disagree) to 5 (completely agree) most respondents rated measures of acceptability and appropriateness high with a mean acceptability value of 4.1 (66% with score ≥ 4) and a mean appropriateness value of 4.0 (73% with score ≥ 4) (Table 7).

**Table 7:** CAQ acceptability and appropriateness (n = 44 subjects who reported reading the CAQ sheet).

Variable	Value*
Acceptability score	4.1 ± 0.8
5	11 (25.0%)
4–5	18 (40.9%)
3–4	13 (29.5%)
<3	2 (4.5%)
Appropriateness score	4.0 ± 0.9
5	12 (27.3%)
4–5	20 (45.5%)
3–4	10 (22.7%)
<3	2 (4.5%)
Acceptability items	
The information sheet meets my approval	4.1 ± 0.9
The information sheet is appealing to me	4.0 ± 0.8
I like the information sheet	4.0 ± 0.9
I welcome the information sheet	4.1 ± 0.9
Appropriateness items	
The information sheet seems fitting	4.0 ± 0.9
The information sheet seems suitable	4.0 ± 0.9
The information sheet seems applicable	4.0 ± 0.9
The information sheet seems like a good match	3.9 ± 1.0
Did not respond	1

*Values are mean ± SD or no. (%); the scoring of the items and the total scores (average of the items) ranged from 1 (completely disagree) to 5 (completely agree).

We ascertained adherence to follow-up information on all enrolled participants (Table 8). Adherence was higher among those who returned the survey compared to non-responders (59% vs. 42%, adjusted difference: 14.5% [95% CI: 4.5, 24.5%], p = 0.005), those who underwent a non-baseline LCS exam compared to a baseline exam (59% vs. 42%, adjusted difference: 15.9% [95% CI: 5.9%, 25.8%], p = 0.002), and those with positive LCS exams compared to Lung-RADS 1–2 (73% vs. 50%, adjusted difference: 23.8% [95% CI: 7.6, 40.0%], p = 0.012). Overall, there was no significant difference in adherence to follow-up comparing the cohort of participants in the CAQ arm vs. the control arm among all eligible participants (54% vs. 50%, adjusted difference: 2.7% [95% CI: -7.0, 12.5%], p = 0.58). However, when stratified by Lung-RADS, adherence was higher in the CAQ arm than the control arm among those with positive exams (90% vs. 42%, adjusted difference: 47.2% [95% CI: 15.8, 78.5%], p = 0.007) but not among those with Lung-RADS 1–2.

**Table 8:** Follow-up adherence rates by groups (n = 386).

		Adherence Rate*		Unadjusted Difference (CAQ–Control)			Adjusted Difference (CAQ–Control)†			
Group	n	CAQ Arm	Control Arm	Mean	(95% CI)	P-value	Mean	(95% CI)	P-value	P-value‡
All eligible	386	112/208 (53.8%)	89/178 (50.0%)	3.8%	(-6.1, 13.8%)	0.45	2.7%	(-7.0, 12.5%)	0.58	
Survey responder	229	75/129 (58.1%)	60/100 (60.0%)	-1.9%	(-14.7, 11.0%)	0.78	-2.1%	(-15.0, 10.8%)	0.75	0.26
Survey non-responder	157	37/79 (46.8%)	29/78 (37.2%)	9.7%	(-5.7, 25.0%)	0.22	9.5%	(-5.6, 24.6%)	0.22	
Lung-RADS 1–2	353	93/187 (49.7%)	84/166 (50.6%)	-0.9%	(-11.3, 9.6%)	0.87	-1.0%	(-11.2, 9.1%)	0.84	**0.008**
Lung-RADS 3–4	33	19/21 (90.5%)	5/12 (41.7%)	48.8%	(18.2, 79.4%)	**0.006**	47.2%	(15.8, 78.5%)	**0.007**	
Baseline LCS exam	161	41/90 (45.6%)	27/71 (38.0%)	7.5%	(-7.7, 22.8%)	0.34	3.4%	(-12.0, 18.7%)	0.66	0.85
Subsequent LCS exam	225	71/118 (60.2%)	62/107 (57.9%)	2.2%	(-10.6, 15.1%)	0.73	2.0%	(-10.7, 14.7%)	0.75	

*Values are no. (%)

†Adjusted for responder status (if all eligible included) and baseline LCS exam (vs. subsequent exam, if both types included)

‡Difference of differences (between the CAQ and control group), calculated using an interaction term between CAQ and row group.

## Discussion

In this study, we conducted a pilot trial of a handout to accompany LCS results to determine the impact on patient-reported understanding of results, next steps in follow-up and distress, and followed participants for adherence to follow-up recommendations. We enrolled sequential patients undergoing LCS in a multisite program over a 6-month period, assessing patient-reported outcomes through a survey with a 59% response rate. While did not find any significant improvement in our primary outcomes, we did find higher report of understanding on specific components of LCS and evidence that adherence was higher in the intervention group with positive index LCS. We also found that the handout was generally considered both acceptable and appropriate. This handout may represent an easy, low-cost and feasible intervention to improve results communication in some patients following LCS, though effective multimodal delivery of education may be needed to successfully improve knowledge and screening adherence.

Previous studies demonstrate that overall follow-up for LCS is poor [2, 4, 26–28], and interventions are needed to ensure follow-up as more than half of all screen-detected lung cancers were diagnosed after the baseline period in LCS clinical trials [29, 30]. Overall, our results show that a hand-out to accompany screening results does not have an overall impact on knowledge, understanding, distress or follow-up when summarized over an entire screening cohort. Of note, we did find that knowledge in the control group was much higher than expected, and higher than our own previous studies of knowledge and understanding of next steps in screening results, which may demonstrate overall improvement in LCS knowledge as screening has become more established, but tempered our ability to affect overall knowledge and understanding through intervention [14]. Even still, our results do suggest that a subset of patients may benefit from this additional information source: those undergoing initial LCS exams, those who interact with patient information sent by mail, and those with the highest risk findings on LCS. While we note some improvements in specific components of understanding in all pariticpants, the magnitude of these improvements was higher in those undergoing baseline LDCT, suggesting this group needs more information following their first LCS exam. We also found that scores on LCS understanding items were higher among the 34% of patients who specifically recalled reading the CAQ handout in the per-procool analysis. That said, the per-protocol results should be interpreted cautiously because the division of the CAQ group into the CAQ-read and CAQ-unread groups is based on participant behavior after the primary intervention group assignment and the CAQ group was relatively small, so there is a higher risk of residual confounding. Finally, we found that adherence to follow-up was significantly higher in the intervention arm among those with positive LDCT results. This may reflect the immediacy of follow-up in this group and the emphasis on follow-up testing based on Lung-RADS categorization in the handout. However, this benefit should be cautiously interpreted given small numbers of participants with positive results and possible unmeasured differences between these groups. It is likely that most of these patients received additional provider-driven recommendations to follow-up, and differential contact with providers may have lead to these findings.

This study represents one of the first published interventions directed at results reporting after LCS. Prior studies demonstrate that many patients find current models of screening results delivery inadequate, particularly when receiving only letter-based communication [20]. Notably, patients demonstrate higher satisfaction with “patient friendly” radiology reports which utilize infographics and patient-centered language [31]. As not all patients have access to their own LDCT reports via patient portals, and radiology reports are not generally designed for patient consumption, we sought to send a patient-friendly information sheet with infographics and patient-centered language to accompany LCS results letters. Although we did not show any difference in primary outcomes, there was some evidence that the intervention may still be useful as part of LCS care given the benefits in specific groups. We may not have seen a difference in knowledge as this was overall high, even in the control group. Patient distress, the other primary outcome, was also low in both groups, similar to prior findings of patients with low-risk lung nodules [32]. Importantly, patient distress was not increased in the group that received the CAQ which should be reassuring that providing additional information does not lead to more distress.

Overall, the CAQ is an intervention that is low-cost and easy to implement and our results suggest this is an acceptable and appropriate communication method to patients. The CAQ can be included in mailings with LCS results to all patients, which contrasts with other interventions which require more costly and intensive programmatic changes, such as program centralization or patient navigation [33, 34]. There may be ways to improve the delivery of the intervention given that only a third of the intervention group reported reading the handout. Delivery through patient portals in EHR, through video or electronic messages, or as an adjunct to in-office discussions may be more effective modes of delivery. The handout may be most useful as part of comprehensive and multi-level interventions to improve results communication and adherence to follow-up.

The strengths of this study include a rigorous approach to study design. We included all patients in a multisite program over a 6 month period, had a consistent randomization scheme with an intention-to-treat analysis, and a 59% response rate to the survey used to delineate patient-reported outcomes. We were able to adjudicate follow-up adherence in all participants enrolled. This study is also based on an intervention with extensive input from patients and providers. Weaknesses of the study include being limited to a single LCS program, small sample size, limitations of a Likert scale measurement, overall limitations in the diversity of the study cohort, and limitations of a mail-only system of delivering the intervention.

In conclusion, this pilot trial of an informational handout to accompany LCS results did not demonstrate improvement in primary outcomes, but may improve individual components of LCS knowledge and results understanding particularly among those who interact with mail and are undergoing an initial LCS, and may improve follow-up among those with positive findings. The impact of the handout may have been tempered by a mail-based delivery; however, such a handout may be a low-cost and feasible intervention to improve screening care among patients undergoing LCS CT. The CAQ handout may represent an important component of a multi-level interventions that can improve screening knowledge and follow-up.

## Supporting information

S1 File The original grant application supporting this project and the Institutional Review Board-approved study protocol. (PDF)

S2 File Detailed regression model output, including all regression coefficients and model fit statistics. (XLSX)

S3 File Supplemental tables comparing PLCOm2012 risk scores between trial groups. (DOCX)
