# Supplementary material for: An alternating-intervention pilot trial on the impact of an informational handout on patient-reported outcomes and follow-up after lung cancer screening
Source: PLoS One. 2024 Apr 10;19(4):e0300352. doi: 10.1371/journal.pone.0300352 (PMC11006146; doi:10.1371/journal.pone.0300352)
Supplement: S1 File — (PDF) [file pone.0300352.s001.pdf]

IRO REC'D SEP 05 2018

SPECIFIC AIMS

Given the 20% reduction in lung cancer mortality associated with **lung cancer screening (LCS)** using annual low-dose chest CT (LDCT) for high risk smokers in the National Lung Screening Trial (NLST),[1] we are now faced with the urgent need to translate this evidence-based strategy to the real-world medical care of patients.[2-8] Successful implementation of LCS requires many factors, including effective communication between patients and healthcare providers before and after the exam. **Our work, supported by the ALA Lung Cancer Discovery (LCD) award that we now seek to renew, has identified important ways to improve communication before patients undergo LDCT** through incorporation of paper and web-based decision aids.[9] We also identified methods to improve patient understanding of the harms and benefits of LCS and identified provider needs and barriers to implementation.[9,10] While adherence to LCS was ~95% in the NLST, emerging studies and our preliminary data suggest it will be lower in clinical settings.[11, 12] Additional challenges to implementation may be encountered in groups who are often poorly represented in clinical trials;[13] the NLST cohort was >90% white, most of whom had a higher education level than the general US population of smokers.[1] Yet, the burden of smoking and lung cancer remain disproportionately high in persons with lower education and lower socioeconomic status (SES).[14, 15]

We now propose moving our research emphasis to identifying ideal processes of care **after the LDCT exam**, as there is little evidence on optimal ways to communicate results of the exam, including describing nodules and other abnormalities (e.g. emphysema), while allaying distress and increasing understanding and adherence to subsequent procedures and annual screening.[11, 16, 17] An ALA LCD renewal award will allow us to focus our work on these critical next steps after the LDCT exam to improve implementation of LCS in diverse populations. Patient-centered communication is important and may influence key outcomes including distress related to results, smoking cessation, and adherence to subsequent testing and screening. Our hypothesis is that a multi-faceted, individualized patient-oriented report of LCS results will facilitate effective communication, reduce patient distress, and support smoking cessation. This report can incorporate statements regarding the likelihood of false-positive findings and personal risk of lung cancer, offer smoking cessation resources, and clearly explain radiology results and follow-up recommendations using terminology that both patients and primary care providers can understand. Ultimately, this report may facilitate increased adherence to evaluation and ongoing LCS, an outcome to be tested in future trials. An innovative aspect of our approach is our focus on patient interaction with radiology reporting and a focus on developing understandable yet comprehensive patient-oriented reports. As patient access in real-time is increasing to their radiology reports through secure web-portals to their electronic health record (EHR),[18, 19] patient understanding of and reaction to the radiology findings may play a key role in optimization of screening and adherence to follow-up after the scan.

We propose the following aims and hypotheses to continue our ALA-supported work to improve the implementation of lung cancer screening, in a diverse range of patients from an academic-affiliated medical center and urban county hospital with a large racial minority and low-income population:

Aim 1. Determine patient-level factors associated with poor adherence to follow-up (repeat CT, other testing) in patients who have previously undergone LCS, using survey methods and EHR data.
H1a: *Poor patient understanding of LCS results will be associated with less adherence to follow-up.*
H1b. *Current smoking, low SES, and minority race will be associated with less adherence to follow-up.*

Aim 2. Explore patient understanding and preferences regarding the methods and information that providers should include in order to communicate the results of LCS in a focus-group based study.
H2a: *Patients will express specific preferences for materials to include when communicating LCS results, including identifying terms for the LCS report that are more understandable and less distressing.*

Aim 3. Compare patient understanding, distress and intention to adhere to follow-up after receipt of a patient-oriented report of LCS results vs. usual care notification in a pilot randomized controlled trial.
H3a. *Patients will find an individualized report of their LCS results to be acceptable, and will report less distress and greater understanding and intention to follow-up with these results compared to usual care notification.*

This project will address key gaps in the ALA "Shared Decision-Making" toolkit for LCS. Our project will inform patient and provider communication regarding nodules and other LCS results such as emphysema, and identify barriers and facilitators of adherence to screening. Our study is innovative and timely, as few prior studies have focused on patient engagement in reading the LDCT radiology report to facilitate patient-oriented communication of results, yet patient interest and access to these reports is increasing.[20, 21] Results of our study can be rapidly translated to improve the clinical care of patients at risk for lung cancer, and will inform the conduct of a future multi-center randomized, controlled trial testing implementation of an individualized, patient-oriented report of LCS results to improve processes and outcomes of LCS.

FHCRC IRB Approval
SEP 17 2018
Document Released Date

A. SIGNIFICANCE

Overview: Successful implementation of **lung cancer screening (LCS)** in the real-world requires many factors, including effective communication between healthcare providers and patients from diverse populations both before and after low-dose chest CT (LDCT). The Centers for Medicare and Medicaid Services (CMS) regulations require shared-decision making incorporating a decision-aid prior to initial LDCT screening.[7] Recent studies, including our ALA supported work (see Preliminary Data), have identified important ways to improve shared-decision making between patients and providers before LCS.[9, 22, 23] Yet, few studies have focused on what happens **after the LDCT** including how to communicate results to patients and how to promote adherence to follow-up. We focus on these significant gaps in knowledge in our ALA grant renewal.

A.1. As LCS is implemented into practice, research in real-world settings with diverse populations is urgently needed to prevent further disparities in care.[6, 24] Nearly 9 million out of 94 million current and former smokers in the US meet NLST criteria,[1, 25] and an estimated 1.5 million or more Americans will have a lung nodule on CT scan annually.[26] Thus, the public health implications of LCS are substantial. Lung cancer is the leading cause of cancer mortality, and incidence is disproportionately elevated in disparity populations.[27] For example, smoking rates remain substantially higher in populations with low socioeconomic status (SES) and with lower levels of education.[28, 29] Disparities in acceptance of cancer screening, quality of cancer care, and cancer mortality already exist along racial and socioeconomic lines.[30-33] As in our previous work, we will address limitations of prior studies by including patients of minority race and low SES in our study.[9]

A.2. Abnormal findings on LDCT are common and LCS can be associated with patient distress; strategies to minimize distress associated with abnormal results are needed.[34] Increased distress after cancer screening may contribute to loss to follow-up.[11, 35] Whereas participants in a LCS clinical trial had transiently increased distress after initial screening,[36, 37] greater distress and confusion are reported in the real-world clinical setting when incidental nodules are detected without the framework of support within a trial.[38-44]

A.3. Quality of provider communication and knowledge can influence distress and other outcomes, yet few studies have investigated how to inform patients of their LCS results.[45-48] Among Veterans with incidentally detected lung nodules, confusion about results and follow-up was common.[38, 48, 49] High quality communication was associated with less distress, and being informed of nodule characteristics was important to patients.[42-44, 48] Providers' own lack of knowledge of nodule evaluation[50-53] may play a key role in poor communication. For breast, cervical and prostate cancer screening, studies show that informed decision-making interventions increase patients' knowledge about need for cancer screening tests and increase adherence to follow-up recommendations.[54] Policies, such as federal mandates, which require standardized communication of results using the Breast Imaging Reporting and Data System (BI-RADS)[55] directly to patients and providers, further support incorporation of this evidence. Pivotal research by our Steering Committee member, Dr. Elmore, in cancer screening mammograms has guided the development of a standardized reporting system for mammography, the Mammography Quality Standards Act enacted by Congress. This Act includes the requirement that mammography facilities provide patients with written results of their mammograms in language that is easy to understand. Our investigative team is poised to apply these lessons learned from mammography to improve the standardization and quality of LCS. No studies have prospectively evaluated how the similar Lung Imaging Reporting and Data System (Lung-RADS)[56] can be combined with nodule-specific strategies[42, 43, 53] for patient-oriented communication of LCS results as we propose.

A.4. As patients have increasing access to their EHR including full radiology results, the LDCT scan report will likely play an important role in patient understanding of LCS results, distress and ultimately adherence. Patients are gaining immediate access to their EHR via web-based portals with many perceived clinical benefits and sense of self-empowerment.[57, 58] Notably, urban, under-served patients of low income and racial/ethnic minorities have a similarly strong interest, though may be offered access less frequently.[59, 60] Patients are also increasingly interested in viewing their complete radiology reports,[20, 61, 62] with the majority (79%) preferring access to radiology reports via patient portals rather than traditional methods.[20] We will be the first to obtain patient feedback regarding use of actual LDCT radiology reports to communicate results of LCS.

A.5. Few studies have investigated adherence to LCS, particularly in a real-world clinical setting.[11] Adherence to cancer screening and follow-up can be lower in those with abnormal results, especially in racial/ethnic minority or low-income populations.[63, 64] In those with incidentally detected nodules, poor communication and distress were associated with less adherence to follow-up,[11, 48] and quality of patient-provider communication generally influences adherence to screening.[65] The role of patient knowledge in impacting distress from and adherence to LCS is uncertain.[66] A greater understanding of factors associated with adherence to LCS follow-up in diverse populations is needed.

A.6. Significance Summary: These studies illustrate the need to develop optimal ways to communicate LCS results, and to understand barriers and motivators to adherence to LCS in diverse populations. Evidence for how best to communicate results to both patients and providers is surprisingly limited, particularly as LCS is initiated in the community with the potential to impact millions of individuals. Our proposal is responsive to published ALA[6] and American Thoracic Society (ATS) research priorities[67, 68] to advance implementation of LCS with attention to improving patient-oriented outcomes of screening. Our proposal will yield directly relevant findings that can be rapidly translated to improve clinical care of patients at risk for lung cancer.

B. INNOVATION: This project seeks to inform and shift current clinical practice by being among the first to:

- Engage patients actively in the process of LCS by having them view and give feedback on the LDCT report; this strategy is relevant and pro-active, as patient access to full radiology reports is growing.[21]
- Evaluate patient-preferred methods to improve communication of LCS results and incorporate provider feedback;[69] results will inform a framework that can become part of an ALA standardized toolkit for LCS.
- Assess patient acceptability, understanding and distress associated with a patient-oriented LCS report.
- Assess barriers and facilitators to adherence to LCS.
- Incorporate patient populations that include a high proportion of minority race/ethnicity and low-income patients who have a high smoking prevalence but have been poorly represented in other studies.

C. APPROACH

C.1. Investigative team: We are a multidisciplinary team with extensive experience in qualitative and quantitative methods, and in the rigorous conduct of clinical research studies. We have expertise in pulmonary medicine and LCS (PI Crothers, co-I Kross, co-I Triplette); primary care (co-I Cole); dissemination and implementation science (co-I Cole); patient communication and psychological outcomes (Kross); developing and adapting health communication materials (collaborator Ko); and integrating smoking cessation with screening and standardization of radiology reporting (collaborator Zeliadt). A Steering Committee composed of Drs. Elmore (primary care, cancer screening) and Pipavath (thoracic radiology) along with Zeliadt and Ko will meet quarterly to provide additional input from key perspectives and assist with formative evaluations on implementation as we aim to develop this project into a future multi-center trial informed by these results.

C.2. Preliminary work: C.2.a. Patients' attitudes regarding LCS and decision aids: A survey and focus group study.[9] In the 1st year (2014-15) of our initial ALA LCD Award we determined, in a low-income, racially diverse population, participants' 1) experience, preferences and reactions to web-based and paper decision aids, and 2) understanding of LCS harms and benefits. We completed 6 focus groups with 45 current or former smokers at Harborview Medical Center (HMC), an urban county hospital. Participants had a mean age of 61; 42% were non-white; 27% had not completed high school; and 50% had an annual income ≤$15,000. Based on pre- and post-surveys, participants' understanding of LCS increased after the groups, particularly of harms including false-positives and extra testing. Five major themes emerged from qualitative analyses: participants 1) were not aware of the purpose of LCS; 2) wanted to know about the benefits and harms; 3) felt physicians need to communicate more effectively; 4) found decision aids helpful and influential for decision-making regarding LCS; and 5) wanted the discussion to be personalized and tailored. These results highlight the diversity of our population, our ability to recruit these participants, and our methodologic expertise, productivity and success of our initial ALA project that we will carry forward in this renewal as we focus on steps after the LDCT exam.

C.2.b. Assessing knowledge, barriers, and facilitators to LCS among primary care and pulmonary providers.[10] To inform implementation of LCS into practice, in the 2nd year (2015-16) of our ALA LCD Award we administered a web-based survey to providers in primary care and pulmonary clinics in the University of Washington (UW) hospital system to assess beliefs, knowledge, current practices, barriers, and facilitators to LCS. Of 196 respondents, 65% were attending physicians and 71% were primary care providers. While the majority (78%) felt that LCS was effective for preventing death from lung cancer, provider knowledge to estimate lung cancer risk and follow-up of nodules was poor. Overall, 50% reported they "don't know" or were "unsure" whether Lung-RADS is important in follow-up of nodules, and 68% needed more information on follow-up recommendations. Providers also identified other barriers (including adherence) at the patient, provider and system levels. These data highlight deficiencies particularly surrounding management of nodules. These data also further underscore the gaps in communication with patients if providers themselves are not clear on recommendations after LDCT. To complement ongoing provider education initiatives, in this renewal we focus on understanding the needs and preferences of patients to inform communication of LCS results.

C.2.c. Adherence to follow-up recommendations after LDCT screening in a real-world setting is low. All patients who undergo LCS in the UW system are tracked using Primordial, a licensed software program to

manage LCS registries (www.PrimordialDesigns.com). We analyzed our current LCS database to determine patient adherence with follow-up; 438 patients who underwent initial screening from 1/2012-6/2016 were included. Adherence to recommended first follow-up was substantially lower than in the NLST. Overall follow-up after initial LDCT was 58% and varied by Lung-RADS score 1-4 (44%, 67%, 75%, 92% respectively). Past barriers to adherence that are likely improved currently include: variability in radiologist recommendations before publication of Lung-RADS; lack of insurance coverage prior to guideline and CMS recommendations;[7, 8] and lack of a robust system to automatically track patients. However, UW began to use Primordial prospectively in 9/2016, and hired a nurse coordinator for the program in 1/2017. Thus, while we anticipate that adherence to follow-up in Aim 1 will be higher as we analyze results of more recent LCS, adherence will likely still be less than 95% seen in the NLST. The work proposed in this project will allow us to investigate factors that can interfere with adherence in real clinical settings, a critical component to successful LCS.

C.2.d. Primary care patient use of web-portals in diverse primary care practices and access to radiology reports. Dr. Cole and colleagues conducted a survey of more than 800 primary care patients across five primary care clinics to assess patient reported use of web-portals.[70] Approximately 39% of patients reported awareness of web-portals for patient-provider communication, and of those 67% reported regular use of web-portals. Dr. Elmore's team led the largest cross-sectional study of patient access to radiology reports to date, involving ~130,000 patients in a major health system and found high patient interest in reading radiology reports online, with 51% of all patients with web-portal access viewing available radiology reports.[21] This high level of interest among patients is corroborated by earlier, smaller studies regarding patient preferences. Among surveyed patients using web-portals, 79% of patients preferred new portal-based methods of receiving results over historical methods, including directly from the referring physician.[20] Similarly, focus groups noted that most patients were dissatisfied with traditional reporting of radiology results, citing delays leading to undue anxiety and distress and little detail when radiologic findings are relayed verbally by a referring physician.[71]

Figure 1. Framework: Improving implementation of lung cancer screening in diverse populations

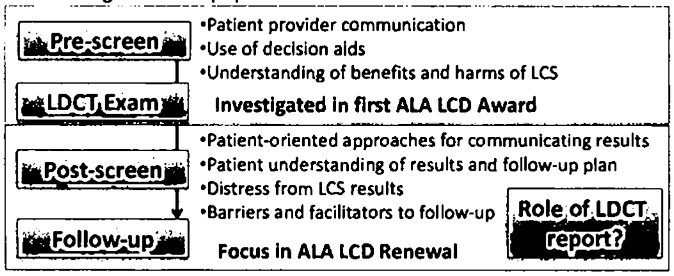

C.3. Framework for assessment of patient-level factors during stages of screening (Figure 1): Our work overall is guided by the Consolidated Framework for Implementation Research (CFIR),[72] which focuses on factors that have been found to be associated with effective implementation. In Aim 1, we will identify potentially modifiable factors in the post-screen stages of LCS associated with adherence. In Aim 2, we will assess patient-specific concerns and preferences to development of individualized, patient-centered approaches to communication, including use of the actual LDCT report. We will test the individualized report and assess acceptability, understanding of and distress with LCS results in Aim 3. With input from the Steering Committee, we will evaluate the outer and inner setting, individual health professionals, and the process of implementation in this pilot trial to prepare us for a larger, multicenter trial to improve implementation of LCS.

C.4.a. Study setting for all Aims: Participants who had LCS will be recruited from primary care and chest clinics affiliated with the University of Washington Medical Center (UWMC), Harborview Medical Center (HMC), and Seattle Cancer Care Alliance (SCCA), [collectively referred to as "UW system"]. We have extensive experience recruiting vulnerable patients for similar studies, including our prior ALA project.[9, 60, 73, 74] We anticipate recruiting ~50% of our participants from HMC, the county hospital, where Drs. Crothers, Cole and Kross are providers. Sites are further described in "Resources," and human subjects in "Assurances."
C.4.b. Patient participants for all Aims: Inclusion criteria: 1) Has undergone LCS, and 2) Able to give informed consent. Exclusion criteria: 1) Cognitive or language limitations (i.e., expressive language limitations, non-English speaking); or 2) Known malignancy other than non-melanoma skin cancer.

Aim 1. Determine patient-level factors associated with poor adherence to follow-up (repeat CT, other testing) in patients who have previously undergone LCS, using survey methods and EHR data.
C.5.a. Rationale: Survey-based study design allows collection of self-reported information from patients to determine patient-level characteristics associated with barriers and facilitators to adherence to follow-up. We will identify LCS patients using Primordial software and collect survey and EHR data to assess whether poor patient-reported understanding or other factors such as smoking, low SES, minority race, or other comorbidities are associated with poor adherence. Additional data will be retrieved from the EHR including actual radiology reports for LDCT results, recommended follow-up, and provider ordered testing. By recruiting patients who have already had LCS, we can efficiently obtain data for analyses in this 2-year award.

C.5.b. Overview of study design and procedures: This is a cross-sectional study. Patients identified from the Primordial LCS registry will be mailed an informed consent and survey to complete. They can choose to either: 1) Complete the paper survey and return it via mail using an enclosed stamped envelope or; 2) Complete the survey via secure on-line access using an identical web-based survey. We will use standard methods to enhance participation rates including a $10 gift card incentive, reminder/thank-you postcards sent 2 weeks after the initial contact, and second packets with surveys sent to non-responders after 4 weeks.[79]

C.5.c. Data Collection: Participants will complete surveys 1-2 months after their planned follow-up per the Lung-RADS score from their index LDCT (e.g.12 months after Lung-RADS 1 or 2; 6 months for Lung-RADS 3).

Table 1. Major variables for Aim 1	Source (reference)
Demographics: age, sex, race/ethnicity; education, income, zip code	Survey, Primordial
Employment and health insurance status	Survey, Primordial
Tobacco use – current or former, pack years	Survey, Primordial
Comorbidities (Charlson comorbidity index)	EHR
Engagement in care (identifies primary care provider, # visits/year)	Survey, EHR
Other prior cancer screening (colonoscopy, mammogram)	Survey, EHR
Understanding of LDCT results (patient report vs. radiologist report)	Survey, EHR
Perception of "being informed" about LDCT results (Likert scale)	Survey[42]
Perception of "being informed" about follow-up plan (Likert scale)	Survey
Perceived risk of lung cancer	Survey[75]
Distress associated with LDCT results (Impact of Event Scale [IES])	Survey[76, 77]
Quality of communication with provider (Consultation Care Measure)	Survey[42, 78]
Patient adherence to provider-ordered follow-up (±30 days of date)	Survey, Primordial[11]

C.5.d. Outcome, predictors and covariates (Table 1): Primary outcome: Adherence to recommendations for follow-up (including LDCT at appropriate interval), defined by Primordial and EHR review (if obtained at UW) or patient report (if not at UW).

C.5.e. Analysis: To determine whether patient adherence (yes/no) to follow-up study (as ordered by the provider) after LDCT differs by patient characteristics and understanding of LDCT results, chi-squared tests will be used to compare categorical variables (such as sex, race/ethnicity, insurance, income, smoking status), t-tests for continuous approximately normally distributed variables (such as age) and Wilcoxon rank-sum tests for non-normally distributed variables (such as pack-years of smoking). Understanding of LDCT (e.g. nodule yes/no) and follow up plan will be dichotomized as good vs. poor. We will evaluate patient's self-assessments of being informed as well as understanding of findings defined by comparing patient-report of results to radiologist report in the EHR. Patient characteristics associated at $p<0.1$ will be assessed in multivariable logistic regression models, adjusting for potential covariates and confounders to determine independent associations. Collinearity will be assessed using Variance Inflation Factors. Potential effect modification (e.g. education, quality of communication with provider) will be assessed by interaction terms and stratification.

C.5.f. Sample size: We will survey patients who have been screened in the 6-15 months prior to the start of the award, and will continue to administer surveys over the first 18 months. In the past 2 years, referrals have grown substantially with 505 patients initiating LCS in 2016 and 2017. We anticipate mailings to ~500 patients, a 40-50% response rate,[42, 43] a range in the primary exposure of 30-60% (poor understanding of LCS results[42, 43]) and a 20-40% range in non-adherence from our preliminary data. Conservatively estimating 200 respondents, we have 80% power to detect an odds ratio of 2.25. This sample size is comparable or larger than studies of incidentally detected nodules that found significant associations.[11, 42, 43]

Aim 2. Explore patient understanding and preferences regarding the methods and information that providers should include in order to communicate the results of LCS in a focus-group based study.

C.6.a. Rationale: There are limited published studies (primarily on incidental nodules[48, 49]) that consider a patient's: 1) understanding of LCS results, and 2) preferences for methods to communicate LCS results. A qualitative approach is particularly appropriate for identifying and describing beliefs and issues that have not been previously examined, and avoids assumptions that could prove to be incorrect if we were to only administer surveys in which items are necessarily constrained and feedback is limited. Patients will form the initial groups, but final groups will include primary care providers to engage in dialogue and obtain feedback.[69]

C.6.b. Overview of study design and procedures: This will be a qualitative study using focus group discussions. A purposive sample of patients who have undergone LCS in the last year will be recruited, making every effort to include patients of diverse race/ethnicity, sex, income, and spanning the eligible age range for LCS. We will pay particular attention to recruiting women as well as men. We will use a combined approach as in our prior study for recruitment,[9] and we will screen using Primordial. Primary care providers in the UW system will be recruited via email or in person to partake in final groups with patients structured as a "circle within a circle" per prior work.[69] After explaining the study, informed signed consent will be obtained from those who will participate. Modest incentives will be offered (e.g. $25 grocery store card, bus voucher, refreshments).

C.6.c. Sample Size: Based on prior experiences, we anticipate that we will reach thematic saturation, the point

at which no new information is gained,[80] by the conclusion of 6-7 focus groups, each with 7-10 participants. The last 2-3 focus groups will include both patients and primary care providers together.

Table 2. Discussion points on LCS results for focus groups
Lung-RADS results: "1. Negative," to "4. Suspicious"[56]
Lung-RADS Category S or "Other" findings (emphysema, coronary calcification)[81]
"What is a Nodule?" handout[82]
Preferences for including and communicating individual lung cancer risk[83-88]
Smoking cessation messages

C.6.d. Data Collection: Focus Groups will be led by the moderator (G Keppel) and note-taker (S Shahrir), and will follow a guide with structured but open-ended questions (see Appendix); they will follow the participants' lead and allow relevant topics not specified to be addressed. We will first pilot the guide[9] and a template of the patient-oriented report, based on Lung-RADS and prior studies,[48, 49, 53] with individual patients. Then, focus groups will start with patients individually viewing de-identified actual LDCT radiology reports, examples of patient letters generated by Primordial for Lung-RADS categories, and completing brief surveys before a group discussion. Surveys will include demographics, education, health literacy, understanding and distress associated with each LDCT report and Primordial letter. As a group, we will assess knowledge and understanding of the materials. We will also discuss other Lung-RADS "Category S" findings such as emphysema; these findings have largely been over-looked but can cause distress and have actionable health consequences.[6, 81] We will ask participants how they would want to be informed of results,[48, 49, 86] review our patient-oriented report for LCS results, and discuss what other patient-individualized information should be included beyond radiology findings, such as individual risk of lung cancer given findings and the "What is a Nodule" handout (Table 2).[89] With Dr. Zeliadt's expertise,[90] we will present smoking cessation messages to identify ways that avoid stigma and encourage adherence.[91, 92] Participants will be asked to discuss the format and content they prefer, why, and suggestions for improvement. We will also elicit barriers and motivators to adherence. We will iteratively refine the report from group to group. The last 2-3 groups will include patients and providers.[69] Groups will last ~70-90 minutes, will be audio-taped with two digital recorders, de-identified and transcribed verbatim by a professional service.

C.6.e. Analysis: To analyze the focus group data, we will use a modified grounded theory approach[93] to describe observations and to construct theory based on the described observations.[94, 95] Steps include: 1) open coding; 2) axial coding; 3) process coding; and 4) selective coding. Findings are used to create a framework in which to understand the factors, context and relationships that enhance or impede understanding and adherence; they will also be used to understand the features of LCS reports that participants identify as most useful, accessible and understandable. Coding will be iterative, with transcripts analyzed after each group so that suggestions or concerns raised by one group can be used to refine the report; this will be further vetted in subsequent groups. The analytic team will include Drs. Crothers, Kross, Cole, and the two interviewers. Two focus group transcripts will be reviewed by the whole analytic team who will develop the coding manual; pairs of analysts will then review transcripts as they are completed with the goal of achieving consensus on the use of codes and contributing to the refinement of the individualized patient-oriented report of LCS results.

Aim 3. Compare patient understanding, distress and intention to adhere to follow-up after receipt of a patient-oriented report of LCS results vs. usual care notification in a pilot randomized controlled trial.
C.7.a. Rationale: We will assess patient understanding, acceptability, and distress with receipt of a patient-oriented report of LCS findings, developed based on Aims 1 and 2, in comparison to usual care. Given the two-year duration of this award, we will assess patient intention to adhere to one-year routine follow-up but actual adherence to shorter-term follow-up. This trial will provide preliminary data in support of a larger clinical trial.

C.7.b. Overview of design: This will be a randomized, controlled pilot study of patients undergoing LCS in the UW system. Patients randomized to the intervention will receive a patient-oriented report based on individual characteristics and results that will replace usual care notification; we will compare this to usual care notification. All participants will receive either notification by mail; surveys will be mailed one week later.

C.7.c. Randomization, intervention and data collection: Participants will be randomized by stratified permuted block randomization with varying block size to receipt of the patient-oriented report of LCS results or usual care notification after LDCT. While there is currently no standard for notification of LCS in the literature, our program has adopted a consistent approach, which includes mailing letters to patients using the templates from Primordial for each Lung-RADS category. Given high uptake of Primordial at other centers across the US, this standard likely reflects usual care across many LCS programs. Providers are expected to telephone patients with LungRADS 3-4 results, but not necessarily LungRADS 1-2. Because those who are initiating LCS may differ from those returning, participants in each arm will be stratified by whether they are undergoing initial vs. follow-up LCS, as well as by clinical site at UW. All patients who have online access to their own EHR may continue to access their data. Providers will be blinded to treatment group. Surveys and consents will be

mailed to patients one week after their LCS report to obtain timely feedback on understanding and reactions to materials received. We will provide paper and secure internet-based options for the survey as in Aim 1.

C.7.d. Outcomes, predictors and covariates: Primary outcomes: **1)** Correct understanding of LDCT (nodules, and category S findings), and needed follow-up (evaluated by concordance between radiologist recommendation and patient statements); 2) Distress, measured using the IES scale;[76, 77] and 3) Acceptability to patients (e.g. "The information was useful"; "The report explained things in terms I could understand."). Secondary outcomes will include: 4) Reported intention and actual adherence to follow-up; 5) Motivation to quit smoking or to remain smoke-free. Other predictors and covariates will be obtained from patients as in Table 1.

C.7.e. Analysis: We will generate descriptive statistics (e.g. mean, standard deviation [SD], median, percent). We will consider differences between groups, adjusting for strata, in generalized linear models for continuous normally distributed variables, in non-parametric rank-based test procedures, and in logistic regression models or stratified contingency tables for binary or categorical variables. We can explore if outcomes differ by site and patient characteristics, including race, SES, and patient-rated quality of provider communication. While we present power for outcomes, a main purpose of a pilot is to test study logistics[96] and gain critical insights into recruitment, randomization, data collection forms, participation rates and barriers/facilitators to implementation.

C.7.f. Sample Size: We will obtain IRB permission to randomize all patients undergoing LCS at UW to this quality improvement intervention (projected ~434 LDCT in 2018, ~1/2 with initial LCS). Assuming 35-40% response rate to surveys, this yields an adequate sample for a pilot study. Using a mean distress (IES) score of 12.9 (SD 15.1),[44] a minimal important difference (MID) of 7 (SD/2),[97] and a balanced design, we have adequate power (80%) with 74 participants in each arm to detect a difference of ≥7 points with alpha of.05. If using categorical data for the outcomes of distress or of poor understanding of results, 80 participants in each group is required, assuming 45% of controls have mild or greater distress (IES≥8)[44] or poor understanding[42, 43] compared to 20-25% of the intervention group, using Pearson's chi-square test for statistical independence.

C.8. Overall Limitations and Alternative Strategies: A critical piece of our work is to ensure that results are relevant across a broad range of patient backgrounds. Though limited to one geographic region, we do not foresee difficulty in achieving our target sample size and are assured of having racially, ethnically, and socioeconomically diverse participants as in our prior work.[9] A potential concern is low response rate from under-served populations; however, we have a large number of patients and a successful track record of recruitment. If we have poor response to surveys, we will obtain IRB permission to call potential participants, and could complete surveys over the telephone with research staff (blinded to study arm in Aim 3). If focus group enrollment is low, we can also recruit participants for one-on-one interviews. Finally, although we have limited our sample to English speakers, this is the primary language for most of our patients, including at HMC.

**Table 3. Deliverables for the ALA Toolkit on LCS**
Immediately relevant and impactful results that will inform clinical care
Patient-preferred methods to improve communication of LCS results
Generalizable recommendations applicable to patients from vulnerable populations at risk for health disparities
Identification of barriers and facilitators to adherence to LCS
Template of an individualized patient-oriented LCS report that can be tested in future trials to assess improvement in patient and provider understanding of results and follow-up, adherence, smoking cessation, and patient-oriented outcomes including distress associated with LCS

C.9. Strengths, Deliverables and Future Directions: We will provide the ALA with data to augment their toolkit with concrete and creative recommendations to improve the implementation of LCS. We will build upon what we have learned in our first ALA LCD to bring a full perspective to improve communication with patients, beginning with a decision to undergo LCS before LDCT is performed to after the exam is completed. Results will be disseminated in the form of presentations at conferences and manuscripts. Our work will establish the basis for future trials, including testing implementation of an individualized LCS report to patients as well as providers. Finally, this ALA LCD would allow Dr. Crothers to continue the mentoring begun during her initial LCD Award, including of Dr. Triplette (now faculty) and Dr. Perrin Romain, who will continue to participate in the scientific investigations proposed. With this renewal, the ALA will also be facilitating and supporting the career development of junior investigators interested in improving lung cancer outcomes.

C.10. Table 4: Timeline and Milestones by Specific Aim over the 2-year funding period

**Aim 1**	Month 1-2: Finalize surveys & obtain IRB approvals for all Aims; use Primordial to identify patients; Months 2-18: administer surveys to patients who have previously undergone LCS; Conduct interim analyses of data obtained by month 8 to inform Aim 3, with final analyses of all data conducted in months 18-21. Anticipated manuscripts: 1) Patient understanding and distress associated with LCS results in a real-world setting; 2) Patient characteristics associated with poor adherence to LCS
**Aim 2**	Months 1-5: Recruit participants and conduct focus groups; Month 6-7: transcription, coding, and analysis of qualitative interviews. Anticipated manuscripts: 1) Patient preferences for reporting and understanding of LCS results
**Aim 3**	Months 6-8: Finalize LCS reports & surveys; Months 9-21: Randomize patients, collect surveys; Months 21-24: clean & analyze data. Anticipated manuscripts: 1) Patient-oriented outcomes associated with LCS report; 2) Implementation assessment

REFERENCES

1. Aberle DR, Adams AM, Berg CD, Black WC, Clapp JD, Fagerstrom RM, Gareen IF, Gatsonis C, Marcus PM, Sicks JD. Reduced lung-cancer mortality with low-dose computed tomographic screening. N Engl J Med. 2011;365(5):395-409.
2. Wender R, Fontham ET, Barrera E, Jr., Colditz GA, Church TR, Ettinger DS, et al. American Cancer Society lung cancer screening guidelines. CA Cancer J Clin. 2013;63(2):107-17. PMCID: 3632634.
3. Bach PB, Mirkin JN, Oliver TK, Azzoli CG, Berry DA, Brawley OW, Byers T, Colditz GA, Gould MK, Jett JR, Sabichi AL, Smith-Bindman R, Wood DE, Qaseem A, Detterbeck FC. Benefits and harms of CT screening for lung cancer: a systematic review. JAMA. 2012;307(22):2418-29. PMCID: 3709596.
4. Jaklitsch MT, Jacobson FL, Austin JH, Field JK, Jett JR, Keshavjee S, MacMahon H, Mulshine JL, Munden RF, Salgia R, Strauss GM, Swanson SJ, Travis WD, Sugarbaker DJ. The American Association for Thoracic Surgery guidelines for lung cancer screening using low-dose computed tomography scans for lung cancer survivors and other high-risk groups. J Thorac Cardiovasc Surg. 2012;144(1):33-8.
5. Matthews AW. Wellpoint to Cover Lung CT Scans for Heavy Smokers. Wall Street Journal. December 1, 2011 December 1, 2011.
6. An Update from the American Lung Association Lung Cancer Screening Committee. Providing Guidance on Lung Cancer Screening to Patients and Physicians. 2015 [updated 2015]; Available from: http://www.lung.org/assets/documents/lung-cancer/lung-cancer-screening-report.pdf.
7. Centers for Medicare & Medicaid Services. Decision memo for screening for lung cancer with low dose computed tomography (LDCT) (CAG-00439N). Available from: https://www.cms.gov/medicare-coverage-database/details/nca-decision-memo.aspx?NCAId=274.
8. Final Recommendation Statement: Lung Cancer: Screening. U.S. Preventive Services Task Force. https://www.uspreventiveservicestaskforce.org/Page/Document/RecommendationStatementFinal/lung-cancer-screening. October 2014 [updated October 2014].
9. Crothers K, Kross EK, Reisch LM, Shahrir S, Slatore C, Zeliadt SB, Triplette M, Meza R, Elmore JG. Patients' Attitudes Regarding Lung Cancer Screening and Decision Aids. A Survey and Focus Group Study. Ann Am Thorac Soc. 2016;13(11):1992-2001.
10. Triplette M, Kross EK, Mann BA, Elmore JG, Slatore CG, Shahrir S, Romine PE, Frederick PD, Crothers K. An Assessment of Primary Care and Pulmonary Provider Perspectives on Lung Cancer Screening. Ann Am Thorac Soc. 2017.
11. Moseson EM, Wiener RS, Golden SE, Au DH, Gorman JD, Laing AD, Deffebach ME, Slatore CG. Patient and Clinician Characteristics Associated with Adherence. A Cohort Study of Veterans with Incidental Pulmonary Nodules. Ann Am Thorac Soc. 2016;13(5):651-9.
12. Hulbert A, Hooker CM, Keruly JC, Brown T, Horton K, Fishman E, et al. Prospective CT screening for lung cancer in a high-risk population: HIV-positive smokers. J Thorac Oncol. 2014;9(6):752-9. PMCID: PMC4023914.
13. Pinsky PF, Ford M, Gamito E, Higgins D, Jenkins V, Lamerato L, Tenorio S, Marcus PM, Gohagan JK. Enrollment of racial and ethnic minorities in the Prostate, Lung, Colorectal and Ovarian Cancer Screening Trial. J Natl Med Assoc. 2008;100(3):291-8.
14. Haiman CA, Stram DO, Wilkens LR, Pike MC, Kolonel LN, Henderson BE, Le Marchand L. Ethnic and racial differences in the smoking-related risk of lung cancer. N Engl J Med. 2006;354(4):333-42.
15. Jamal A, Homa DM, O'Connor E, Babb SD, Caraballo RS, Singh T, Hu SS, King BA. Current cigarette smoking among adults - United States, 2005-2014. MMWR Morb Mortal Wkly Rep. 2015;64(44):1233-40.
16. Montes U, Seijo LM, Campo A, Alcaide AB, Bastarrika G, Zulueta JJ. Factors determining early adherence to a lung cancer screening protocol. Eur Respir J. 2007;30(3):532-7.
17. Wildstein KA, Faustini Y, Yip R, Henschke CI, Ostroff JS. Longitudinal predictors of adherence to annual follow-up in a lung cancer screening programme. J Med Screen. 2011;18(3):154-9.
18. Henshaw D, Okawa G, Ching K, Garrido T, Qian H, Tsai J. Access to Radiology Reports via an Online Patient Portal: Experiences of Referring Physicians and Patients. J Am Coll Radiol. 2015;12(6):582-6 e1.
19. Lee CI, Langlotz CP, Elmore JG. Implications of Direct Patient Online Access to Radiology Reports Through Patient Web Portals. J Am Coll Radiol. 2016;13(12 Pt B):1608-14.
20. Johnson AJ, Easterling D, Nelson R, Chen MY, Frankel RM. Access to radiologic reports via a patient portal: clinical simulations to investigate patient preferences. J Am Coll Radiol. 2012;9(4):256-63.
21. Miles RC, Hippe DS, Elmore JG, Wang CL, Payne TH, Lee CI. Patient Access to Online Radiology Reports: Frequency and Sociodemographic Characteristics Associated with Use. Acad Radiol. 2016;23(9):1162-9.

22. Mishra SI, Sussman AL, Murrietta AM, Getrich CM, Rhyne R, Crowell RE, Taylor KL, Reifler EJ, Wescott PH, Saeed AI, Hoffman RM. Patient Perspectives on Low-Dose Computed Tomography for Lung Cancer Screening, New Mexico, 2014. Prev Chronic Dis. 2016;13:E108. PMCID: PMC4993119.
23. Schapira MM, Aggarwal C, Akers S, Aysola J, Imbert D, Langer C, Simone CB, 2nd, Strittmatter E, Vachani A, Fraenkel L. How Patients View Lung Cancer Screening. The Role of Uncertainty in Medical Decision Making. Ann Am Thorac Soc. 2016;13(11):1969-76.
24. Aberle DR, Henschke CI, McLoud TC, Boiselle PM. Expert opinion: barriers to CT screening for lung cancer. J Thorac Imaging. 2012;27(4):208.
25. Katki HA, Kovalchik SA, Berg CD, Cheung LC, Chaturvedi AK. Development and Validation of Risk Models to Select Ever-Smokers for CT Lung Cancer Screening. JAMA. 2016;315(21):2300-11. PMCID: PMC4899131.
26. Gould MK, Tang T, Liu IL, Lee J, Zheng C, Danforth KN, Kosco AE, Di Fiore JL, Suh DE. Recent Trends in the Identification of Incidental Pulmonary Nodules. Am J Respir Crit Care Med. 2015;192(10):1208-14.
27. Di Cesare M, Khang YH, Asaria P, Blakely T, Cowan MJ, Farzadfar F, Guerrero R, Ikeda N, Kyobutungi C, Msyamboza KP, Oum S, Lynch JW, Marmot MG, Ezzati M. Inequalities in non-communicable diseases and effective responses. Lancet. 2013;381(9866):585-97.
28. Hiscock R, Bauld L, Amos A, Fidler JA, Munafo M. Socioeconomic status and smoking: a review. Ann N Y Acad Sci. 2012;1248:107-23.
29. Current cigarette smoking among adults - United States, 2011. MMWR Morb Mortal Wkly Rep. 2012;61(44):889-94.
30. Elmore JG, Nakano CY, Linden HM, Reisch LM, Ayanian JZ, Larson EB. Racial inequities in the timing of breast cancer detection, diagnosis, and initiation of treatment. Med Care. 2005;43(2):141-8.
31. Martires KJ, Kurlander DE, Minwell GJ, Dahms EB, Bordeaux JS. Patterns of cancer screening in primary care from 2005 to 2010. Cancer. 2013.
32. Clegg LX, Li FP, Hankey BF, Chu K, Edwards BK. Cancer survival among US whites and minorities: a SEER (Surveillance, Epidemiology, and End Results) Program population-based study. Arch Intern Med. 2002;162(17):1985-93.
33. Albano JD, Ward E, Jemal A, Anderson R, Cokkinides VE, Murray T, Henley J, Liff J, Thun MJ. Cancer mortality in the United States by education level and race. J Natl Cancer Inst. 2007;99(18):1384-94.
34. Slatore CG, Sullivan DR, Pappas M, Humphrey LL. Patient-centered outcomes among lung cancer screening recipients with computed tomography: a systematic review. J Thorac Oncol. 2014;9(7):927-34.
35. O'Donnell S, Goldstein B, Dimatteo MR, Fox SA, John CR, Obrzut JE. Adherence to mammography and colorectal cancer screening in women 50-80 years of age the role of psychological distress. Womens Health Issues. 2010;20(5):343-9.
36. van den Bergh KA, Essink-Bot ML, Borsboom GJ, Scholten ET, van Klaveren RJ, de Koning HJ. Long-term effects of lung cancer computed tomography screening on health-related quality of life: the NELSON trial. Eur Respir J. 2011;38(1):154-61.
37. van den Bergh KA, Essink-Bot ML, Borsboom GJ, Th Scholten E, Prokop M, de Koning HJ, van Klaveren RJ. Short-term health-related quality of life consequences in a lung cancer CT screening trial (NELSON). Br J Cancer. 2010;102(1):27-34. PMCID: 2813757.
38. Slatore CG, Press N, Au DH, Curtis JR, Wiener RS, Ganzini L. What the heck is a "nodule"? A qualitative study of veterans with pulmonary nodules. Ann Am Thorac Soc. 2013;10(4):330-5. PMCID: PMC3780978.
39. Mazzone PJ, Obuchowski N, Fu AZ, Phillips M, Meziane M. Quality of life and healthcare use in a randomized controlled lung cancer screening study. Ann Am Thorac Soc. 2013;10(4):324-9.
40. Wiener RS, Gould MK, Woloshin S, Schwartz LM, Clark JA. 'The thing is not knowing': patients' perspectives on surveillance of an indeterminate pulmonary nodule. Health Expect. 2012.
41. Byrne MM, Weissfeld J, Roberts MS. Anxiety, fear of cancer, and perceived risk of cancer following lung cancer screening. Med Decis Making. 2008;28(6):917-25.
42. Slatore CG, Golden SE, Ganzini L, Wiener RS, Au DH. Distress and patient-centered communication among veterans with incidental (not screen-detected) pulmonary nodules. A cohort study. Ann Am Thorac Soc. 2015;12(2):184-92. PMCID: 4342836.
43. Freiman MR, Clark JA, Slatore CG, Gould MK, Woloshin S, Schwartz LM, Wiener RS. Patients' Knowledge, Beliefs, and Distress Associated with Detection and Evaluation of Incidental Pulmonary Nodules for Cancer: Results from a Multicenter Survey. J Thorac Oncol. 2016;11(5):700-8. PMCID: PMC4851914.

44. Slatore CG, Wiener RS, Golden SE, Au DH, Ganzini L. Longitudinal Assessment of Distress among Veterans with Incidental Pulmonary Nodules. Ann Am Thorac Soc. 2016;13(11):1983-91.
45. Villani J, Mortensen K. Patient-provider communication and timely receipt of preventive services. Prev Med. 2013;57(5):658-63.
46. Allen JD, Shelton RC, Harden E, Goldman RE. Follow-up of abnormal screening mammograms among low-income ethnically diverse women: findings from a qualitative study. Patient Educ Couns. 2008;72(2):283-92.
47. Carcaise-Edinboro P, Bradley CJ. Influence of patient-provider communication on colorectal cancer screening. Med Care. 2008;46(7):738-45.
48. Wiener RS, Gould MK, Woloshin S, Schwartz LM, Clark JA. What do you mean, a spot?: A qualitative analysis of patients' reactions to discussions with their physicians about pulmonary nodules. Chest. 2013;143(3):672-7. PMCID: 3590883.
49. Sullivan DR, Golden SE, Ganzini L, Hansen L, Slatore CG. 'I still don't know diddly': a longitudinal qualitative study of patients' knowledge and distress while undergoing evaluation of incidental pulmonary nodules. NPJ Prim Care Respir Med. 2015;25:15028. PMCID: 4532158.
50. Tanner NT, Aggarwal J, Gould MK, Kearney P, Diette G, Vachani A, Fang KC, Silvestri GA. Management of Pulmonary Nodules by Community Pulmonologists: A Multicenter Observational Study. Chest. 2015;148(6):1405-14. PMCID: PMC4665735.
51. Wiener RS, Slatore CG, Gillespie C, Clark JA. Pulmonologists' Reported Use of Guidelines and Shared Decision-making in Evaluation of Pulmonary Nodules: A Qualitative Study. Chest. 2015;148(6):1415-21. PMCID: PMC4665736.
52. Golden SE, Wiener RS, Sullivan D, Ganzini L, Slatore CG. Primary Care Providers and a System Problem: A Qualitative Study of Clinicians Caring for Patients With Incidental Pulmonary Nodules. Chest. 2015;148(6):1422-9. PMCID: PMC4665737.
53. Woloshin S, Schwartz LM, Dann E, Black WC. Using radiology reports to encourage evidence-based practice in the evaluation of small, incidentally detected pulmonary nodules. A preliminary study. Ann Am Thorac Soc. 2014;11(2):211-4.
54. Briss P, Rimer B, Reilley B, Coates RC, Lee NC, Mullen P, et al. Promoting informed decisions about cancer screening in communities and healthcare systems. Am J Prev Med. 2004;26(1):67-80.
55. American College of Radiology. The ACR breast imaging reporting and data system (BI-RADS) [web source]. November 11, 2003.; 2003 [updated 2003]; Available from: http://www.acr.org/departments/stand_accred/birads/contents.html.
56. American College of Radiology. Lung CT Screening Reporting and Data System (Lung-RADS). Available from: https://www.acr.org/Quality-Safety/Resources/LungRADS.
57. Delbanco T, Walker J, Bell SK, Darer JD, Elmore JG, Farag N, Feldman HJ, Mejilla R, Ngo L, Ralston JD, Ross SE, Trivedi N, Vodicka E, Leveille SG. Inviting patients to read their doctors' notes: a quasi-experimental study and a look ahead. Ann Intern Med. 2012;157(7):461-70. PMCID: PMC3908866.
58. Walker J, Leveille SG, Ngo L, Vodicka E, Darer JD, Dhanireddy S, Elmore JG, Feldman HJ, Lichtenfeld MJ, Oster N, Ralston JD, Ross SE, Delbanco T. Inviting patients to read their doctors' notes: patients and doctors look ahead: patient and physician surveys. Ann Intern Med. 2011;155(12):811-9. PMCID: 3772715.
59. Peacock S, Reddy A, Leveille SG, Walker J, Payne TH, Oster NV, Elmore JG. Patient portals and personal health information online: perception, access, and use by US adults. J Am Med Inform Assoc. 2016.
60. Dhanireddy S, Walker J, Reisch L, Oster N, Delbanco T, Elmore JG. The urban underserved: attitudes towards gaining full access to electronic medical records. Health Expect. 2012. PMCID: 3469742.
61. Johnson AJ, Easterling D, Williams LS, Glover S, Frankel RM. Insight from patients for radiologists: improving our reporting systems. J Am Coll Radiol. 2009;6(11):786-94.
62. Direct Access: Online Portals Give Patients Easy Access to Radiology Reports. American College of Radiology; 2013 [updated 2013]; Available from: http://www.acr.org/News-Publications/News/News-Articles/2013/ACR-Bulletin/201306-Direct-Access.
63. Taylor KL, Shelby R, Gelmann E, McGuire C. Quality of life and trial adherence among participants in the prostate, lung, colorectal, and ovarian cancer screening trial. J Natl Cancer Inst. 2004;96(14):1083-94.
64. Ford ME, Havstad SL, Flickinger L, Johnson CC. Examining the effects of false positive lung cancer screening results on subsequent lung cancer screening adherence. Cancer Epidemiol Biomarkers Prev. 2003;12(1):28-33.

65. Peterson EB, Ostroff JS, DuHamel KN, D'Agostino TA, Hernandez M, Canzona MR, Bylund CL. Impact of provider-patient communication on cancer screening adherence: A systematic review. Prev Med. 2016;93:96-105.
66. Slatore CG, Wiener RS. Pulmonary Nodules: A Small Problem for Many, Severe Distress for Some, and How to Communicate About It. Chest. 2017.
67. Mazzone P, Powell CA, Arenberg D, Bach P, Detterbeck F, Gould MK, Jaklitsch MT, Jett J, Naidich D, Vachani A, Wiener RS, Silvestri G. Components necessary for high-quality lung cancer screening: American College of Chest Physicians and American Thoracic Society Policy Statement. Chest. 2015;147(2):295-303. PMCID: PMC4502754.
68. Slatore CG, Horeweg N, Jett JR, Midthun DE, Powell CA, Wiener RS, Wisnivesky JP, Gould MK. A.T.S. Ad Hoc Committee on Setting a Research Framework for Pulmonary Nodule Evaluation. An Official American Thoracic Society Research Statement: A Research Framework for Pulmonary Nodule Evaluation and Management. Am J Respir Crit Care Med. 2015;192(4):500-14.
69. Gallagher TH, Waterman AD, Ebers AG, Fraser VJ, Levinson W. Patients' and physicians' attitudes regarding the disclosure of medical errors. JAMA. 2003;289(8):1001-7.
70. Bauer AM, Rue T, Munson SA, Ghomi RH, Keppel GA, Cole AM, Baldwin LM, Katon W. Patient-oriented health technologies: Patients' perspectives and use. J Mob Technol Med. 2017;6(2):1-10. PMCID: PMC5603303.
71. Johnson AJ, Frankel RM, Williams LS, Glover S, Easterling D. Patient access to radiology reports: what do physicians think? J Am Coll Radiol. 2010;7(4):281-9.
72. Damschroder LJ, Aron DC, Keith RE, Kirsh SR, Alexander JA, Lowery JC. Fostering implementation of health services research findings into practice: a consolidated framework for advancing implementation science. Implement Sci. 2009;4:50. PMCID: PMC2736161.
73. Geiger AM, West CN, Nekhlyudov L, Herrinton LJ, Liu IL, Altschuler A, Rolnick SJ, Harris EL, Greene SM, Elmore JG, Emmons KM, Fletcher SW. Contentment with quality of life among breast cancer survivors with and without contralateral prophylactic mastectomy. J Clin Oncol. 2006;24(9):1350-6.
74. Linden HM, Reisch LM, Hart A, Jr., Harrington MA, Nakano C, Jackson JC, Elmore JG. Attitudes toward participation in breast cancer randomized clinical trials in the African American community: a focus group study. Cancer Nurs. 2007;30(4):261-9.
75. Carter-Harris L, Slaven JE, 2nd, Monohan P, Rawl SM. Development and Psychometric Evaluation of the Lung Cancer Screening Health Belief Scales. Cancer Nurs. 2016. PMCID: PMC4890636.
76. Joseph S. Psychometric evaluation of Horowitz's Impact of Event Scale: a review. J Trauma Stress. 2000;13(1):101-13.
77. Vodermaier A, Linden W, Siu C. Screening for emotional distress in cancer patients: a systematic review of assessment instruments. J Natl Cancer Inst. 2009;101(21):1464-88. PMCID: PMC3298956.
78. Hudon C, Fortin M, Haggerty JL, Lambert M, Poitras ME. Measuring patients' perceptions of patient-centered care: a systematic review of tools for family medicine. Ann Fam Med. 2011;9(2):155-64. PMCID: PMC3056864.
79. Dillman DA. Mail and Internet Surveys: The Tailored Design Method. New York: John Wiley & Sons, Inc; 2000.
80. The Handbook of Qualitative Research. Denzin N, Lincoln Y, editors. Thousand Oaks CA: Sage Publications; 1994.
81. Sather P, Dicks D, Killam J, Curtis A, Detterbeck F, Pisani M, Tanoue LT. The Impact of Structured Reading for Incidental Findings Identified on Lung Cancer Screening Low-Dose Computed Tomography (LDCT) Scans. Am J Respir Crit Care Med. 2016;193:A1278.
82. Slatore CG, Wiener RS, Laing AD. What is a Lung Nodule? Am J Respir Crit Care Med. 2016;193(7):P11-2.
83. McCaffery KJ, Dixon A, Hayen A, Jansen J, Smith S, Simpson JM. The influence of graphic display format on the interpretations of quantitative risk information among adults with lower education and literacy: a randomized experimental study. Med Decis Making. 2012;32(4):532-44.
84. Hildon Z, Allwood D, Black N. Impact of format and content of visual display of data on comprehension, choice and preference: a systematic review. Int J Qual Health Care. 2012;24(1):55-64.
85. Dolan JG, Iadarola S. Risk communication formats for low probability events: an exploratory study of patient preferences. BMC Med Inform Decis Mak. 2008;8:14. PMCID: 2330036.

86. Leekha S, Thomas KG, Chaudhry R, Thomas MR. Patient preferences for and satisfaction with methods of communicating test results in a primary care practice. Jt Comm J Qual Patient Saf. 2009;35(10):497-501.
87. Zipkin DA, Umscheid CA, Keating NL, Allen E, Aung K, Beyth R, Kaatz S, Mann DM, Sussman JB, Korenstein D, Schardt C, Nagi A, Sloane R, Feldstein DA. Evidence-based risk communication: a systematic review. Ann Intern Med. 2014;161(4):270-80.
88. Fagerlin A, Zikmund-Fisher BJ, Ubel PA. Helping patients decide: ten steps to better risk communication. J Natl Cancer Inst. 2011;103(19):1436-43. PMCID: PMC3218625.
89. Peters E, Hart PS, Fraenkel L. Informing patients: the influence of numeracy, framing, and format of side effect information on risk perceptions. Med Decis Making. 2011;31(3):432-6.
90. Zeliadt SB, Heffner JL, Sayre G, Klein DE, Simons C, Williams J, Reinke LF, Au DH. Attitudes and Perceptions About Smoking Cessation in the Context of Lung Cancer Screening. JAMA Intern Med. 2015;175(9):1530-7.
91. Quaife SL, Marlow LA, McEwen A, Janes SM, Wardle J. Attitudes towards lung cancer screening in socioeconomically deprived and heavy smoking communities: informing screening communication. Health Expect. 2016.
92. Carter-Harris L, Ceppa DP, Hanna N, Rawl SM. Lung cancer screening: what do long-term smokers know and believe? Health Expect. 2015. PMCID: PMC4919238.
93. Bradley EH, Curry LA, Devers KJ. Qualitative data analysis for health services research: developing taxonomy, themes, and theory. Health Serv Res. 2007;42(4):1758-72. PMCID: 1955280.
94. Glaser B, Strauss A. Discovery of Grounded Theory. Chicago: Adline Publishing Company; 1967.
95. Strauss A, Corbin J. Basics of Qualitative Research: Techniques and Procedures for Developing Grounded Theory. Thousand Oaks: Sage Publications; 1998.
96. Kistin C, Silverstein M. Pilot Studies: A Critical but Potentially Misused Component of Interventional Research. JAMA. 2015;314(15):1561-2. PMCID: PMC4917389.
97. Norman GR, Sloan JA, Wyrwich KW. Interpretation of changes in health-related quality of life: the remarkable universality of half a standard deviation. Med Care. 2003;41(5):582-92.

ASSURANCES

(1) HUMAN SUBJECTS

Training and Certification in Human Subjects Research
All personnel will be required to provide written evidence documenting education in the protection of human subjects. Personnel who have not previously completed human subject training will be required to complete the CITI Basic Course in Human Subject Protections. This is an online self-paced course that proceeds in a stepwise fashion, module by module with the choice of either a biomedical or social/behavioral focus. The web address for this course is: http://citiprogram.org.

1. Risks to the subjects
1.a. Human subjects' involvement and characteristics: For all aims, we will recruit patients from the primary care and pulmonary clinics affiliated with Harborview Medical Center (HMC), the University of Washington (UW) Medical Center (UWMC), and the Seattle Cancer Care Alliance (SCCA) Early Lung Cancer Detection and Prevention Clinic, all in Seattle, WA. We anticipate mailing surveys to approximately 500 participants with 200 respondents for Aim 1; enrolling 60-70 participants for Aim 2; and mailing surveys to approximately 430 participants with 160 respondents for Aim 3. Similar inclusion and exclusion criteria will be used for all aims. Inclusion criteria: 1) Completed lung cancer screening (LCS) with at least an initial low dose chest CT (LDCT), and 2) Able to give informed consent. Exclusion criteria: 1) Cognitive or language limitations (i.e., expressive language limitations, non-English speaking); or 2) Known malignancy other than non-melanoma skin cancer. We anticipate surveys will take approximately 20 minutes to complete. Focus groups will require approximately 70-90 minute. Patients will range in age, and demographic characteristics. No patients will be excluded based on demographic characteristics.

1.b. Sources of materials: Sources of data will include: 1) Electronic health record (EHR) and Primordial lung cancer registry database; 2) Surveys; 3) Digitally recorded focus group sessions or interviews; 4) Anonymous transcripts of focus groups.

1.c. Potential risks: The potential risks for harm from this research include those that may result from invasion of privacy, breach of confidentiality, distress or anxiety, and lost time. This study will be designed specifically to minimize risks to subjects. We will implement and strictly adhere to security measures designed to protect participant confidentiality and ensure data security.
EHR and Primordial database review: The primary risk associated with EHR and Primordial review is invasion of privacy and breach of confidentiality.
Surveys: The potential risks are minor, and are primarily related to the stress of completing surveys and the risk to privacy. Surveys are voluntary, and subjects are free to skip any questions they do not wish to answer. Subjects are informed in consent forms that completing the surveys may cause emotional distress by causing them to think about symptoms and conditions that are unpleasant.
Qualitative interviews are digitally recorded sessions: Participants will be made aware during the informed consent process that focus group sessions will be digitally recorded and that the nature of these group will involve participants speaking about LCS. No participant full names will be recorded on the tapes and the transcripts will be coded by participant number rather than name in order to protect participant confidentiality. Efforts will be made to minimize the burden of stress for all participants. During qualitative data collection, focus group leaders will assure participants that they can, at any time during the group, refuse to answer any question, and at any time elect to withdraw from the study. Further, they will assure participants that the data will be anonymous, and that their participation or lack thereof will not be reported to their peers or providers.

1.d. Treatment alternatives: No alternative. Participants may decline to partake in this study.

2. Adequacy of protection against risks

2.a. Recruitment and informed consent:
Aim 1. Patients who have undergone LCS at UW will be identified from the Primordial LCS registry. They will be mailed an informed consent form and survey to complete. They can choose to either: 1) Complete the paper survey and return it via mail using the enclosed stamped envelope or; 2) Complete the consent and survey via secure on-line access using an identical web-based survey. As in our other studies, return receipt of surveys via mail but without a signed consent will imply consent. We will send reminder/thank-you postcards 2 weeks after initial contact, and 2nd packets with surveys sent to non-responders after 4 weeks.

Aim 2. We have extensive experience recruiting patients from these sites for similar studies. We will use a combined approach to recruit patients as in our prior ALA supported work. First, we will ask providers to

identify potentially eligible participants at the time of their clinic visit. Second, we will screen Primordial to identify potentially eligible subjects and obtain IRB permission to telephone these participants to explain the study and invite them to enroll. Lastly, we will also advertise the study through fliers in the clinic. Interested participants will be phoned or may be approached in clinic by the Research Coordinator. Primary care provider participants in the UW system will be recruited via email or in person; we will recruit providers from each of the sites within the UW system. After explaining study procedures, informed signed consent will be obtained from all participants. We will work with the clinic directors and staff to ensure minimal disruption to clinic flow or patient care.

Aim 3. Patients who are referred for LCS at UW will be identified via the nurse coordinator and Primordial registry. We will obtain IRB permission for a waiver of informed consent to send an individualized report of LCS results to patients randomized to the intervention arm vs. usual care over approximately a one-year period. The report may also include the "What is a nodule" patient education handout developed for the American Thoracic Society, depending on feedback about the usefulness of this handout from patients in Aim 2. The report will be mailed in place of the usual care notification, which consists only of a form letter generated by Primordial for each LungRADS category. One week after patients receive their individualized or usual care report of LCS results, we will mail surveys and consent forms to patients, following similar procedures as in Aim 1.

2.b. Protection against risk: To maintain confidentiality of all study materials, we will record names and contact information in a database for the purpose of mailing of surveys and focus group scheduling. On all other materials we will use unique identification numbers and identifying information will be deleted from the audiotapes. Identifying information will be kept separate from transcribed focus groups in locked file cabinets and password protected databases. All data, including focus group transcripts, will be stored in a secure, project-specific, password-protected folder on the UW server on a drive specifically designed to house and protect research data. Identifying information will be deleted at the earliest possible date.

In order to maintain the quality of the data, we will implement systematic data collection, quality control, and data management procedures including: 1) specification and use of concise protocols for audio-taping, transcribing and coding; 2) rigorous training, certification and periodic re-training of study staff with on-going monitoring of adherence to qualitative and quantitative, survey-based data collection and handling protocols; 3) validation and verification of all tracking data collected through use of computerized data entry systems; and 4) twice monthly meetings and progress reports to provide specific, well-documented feedback to study staff concerning potential difficulties as well as follow-up to ensure that problems are resolved quickly.
Surveys: Survey data will be collected without individual identifiers of participants, such as recording only age and not actual date of birth, and will not be labeled with medical record numbers.
Digitally recorded sessions: All appropriate actions will be taken by staff members in order to minimize the risks associated with loss of confidentiality. Audio files will be coded by number and will be erased.
Confidentiality: Numerous steps will be taken to protect confidentiality as outlined. Individually identifiable health information will be protected in accordance with the Health Insurance Portability and Accountability Act (HIPAA) of 1996. All research personnel will be trained on Institutional Review Board (IRB) and HIPAA procedures.

3. Potential benefits of the proposed research to the subjects and others
There is no guaranteed individual benefit from participating in this study. Participants will contribute to our understanding of factors that may improve understanding, patient-centered outcomes and adherence to LCS.

4. Importance of the knowledge to be gained
There is limited information to date on the barriers and facilitators to adherence to LCS in diverse settings, and limited data on optimal ways to communicate results of LCS from patient perspectives. The proposed study will provide valuable information regarding these factors, and will serve to advance our understanding of how to better implement lung cancer screening.

5. Inclusion of Women and Minorities
We anticipate enrolling approximately 40-50% minorities as in our prior ALA study, and will actively recruit women.

6. Inclusion of Children
Children will not be included, as lung cancer screening is only indicated for older adults.

(2) ANIMAL USE AND JUSTIFICATION: No animals will be used in this project.

(3) BIOHAZARDS MATERIALS: No biohazards will be used in this project.

APPENDIX: AMERICAN LUNG ASSOCIATION LUNG CANCER DISCOVERY GRANT (PI Crothers)

FOCUS GROUP INTERVIEW GUIDE

Introduction— purpose and ground rules

My name is ________________ and I will be leading this session to talk with you about lung cancer screening.

Thank you so much for being here today. We have received a grant from the American Lung Association to find out good ways that doctors can use in talking with their patients about the results of lung cancer screening. You are all here today because have recently undergone a low-dose CT for lung cancer screening, and we are interested in your opinions on how doctors can best communicate the results of these CT tests to their patients.

INDIVIDUAL INTRODUCTIONS

Before we get started, has everyone signed the consent to be audiotaped? This tape is used to summarize our discussion. No one will listen to it but members of the research team.

A few ground rules:
We are not looking for you to all agree; in fact, we don't expect you to agree with each other. The screening test for lung cancer is an imaging study called a low-dose chest CT scan. You all recently went through one of these tests. We want to learn ways to communicate better with patients about how to talk about the results so that they are easy to understand. We expect there will be a range of experiences and we expect to learn from the variety --and tailor our program to address what patients worry about and what they need to know.

If any questions come up about your own recommendations, please discuss your questions with your doctor or healthcare provider -- Please do not make any screening or treatment decision based on our group discussion.

Just a few more rules:
If you tend to talk a lot, I hope you won't mind if I ask you to stop so that we can hear from everyone. If you are shy, I hope you'll speak up and participate.

We encourage you to share your opinions with the group but please avoid side conversations while others are speaking. We are taping this group so we can hear from everyone, and when there are side conversations it interferes with the clarity of the taping.

A reminder: please turn off your cell phone.

Are there any questions before we get started?

1. Everyone here has had a lung cancer screening CT exam. What is the ONE thing you remember most about your CT exam?

2. Most of you have received the results of your LDCT. What was it like waiting to hear the results? What is the ONE thing you remember most about getting the results of your CT? What was it like hearing the results? Are you happy with your decision to be screened? Why, or why not?

3. REVIEW OF MOCK RADIOLOGY REPORT BASED ON LUNG-RADS: While we were waiting for this focus group discussion to begin, we gave you each time to view actual radiology reports from different LDCT scans. Let's walk through this example – not one of your own reports – together, and I'd like to hear from you how well you understand the various pieces of the report.

a. What were the results of this scan, and what do the results mean for the patient?
b. What parts of the report make sense to you, and please explain to the group what those pieces mean.
c. What parts are confusing to you, and please explain to the group what you find confusing.
d. Is it a good idea for patients to see the radiology report?
e. Is it a good idea for patients to see the pictures from the radiology test itself?

4. REVIEW OF HOW PATIENTS WERE INFORMED OF THEIR OWN RESULTS: Think about when you had your CT scan.

a. Did your doctor show you your radiology report? Images?
b. Did your doctor go over the results with you? How? Did they explain in words, show pictures or pamphlets, etc.?

5. When your doctor went over the results of your LDCT with you, what parts of the discussion did you understand well? How were those parts explained to you? What was it that you liked about your doctor's communication? Did your doctor talk to you about your risk of lung cancer? Did you want to know what your risk of lung cancer was based on your LDCT?

6. When your doctor went over the results of your LDCT with you, what parts were confusing to you? How were those parts explained to you? What parts were distressing to you? What about their communication didn't work well? What could your doctor have done differently to increase your understanding of the results? What could your doctor have done to make you less worried?

7. Pass out "What is a nodule" information sheet. I'm going to show you some of the issues to consider about the possible results of lung cancer screening using pamphlets that your doctor could show you. We want you to tell us your thoughts about whether this helps you understand what your possible test results would be. [Provide each participant with a copy of the pamphlets]

Based on our discussion thus far and seeing this pamphlet, do you think you understand the possible results of a lung cancer screening test?

a. What could the results of a LDCT be?
b. What would the results mean to the patient?
c. Did the pamphlet help you?
d. How do you think the pamphlet could be improved?
e. Do you want to know how frequently each type of result can occur, as illustrated in the picture, or just what the possible results could be?
f. Would you want your doctor's office to use a pamphlet like this when explaining your results? What other methods of explaining could be used?

8. After hearing more about the results of lung cancer screening, have you decided to undergo a repeat LDCT in the future when recommended by your doctor? – Why/why not?

Thank you so much for participating. This has been a really useful experience and you have provided a lot of very valuable information. Do you have any final comments?

IRO REC'D OCT 17 2018

Community-based Outcomes and Adherence in Lung cancer Screening (COALS)

November 1, 2017

1. Background and Rationale

1.1. Introduction

The National Lung Screening Trial (NLST), published in 2011, demonstrated a 20% lung cancer mortality risk reduction from annual low-dose computed tomography (LDCT) compared to chest radiography in a defined cohort of high-risk smokers.[1] On the basis of this trial, the United States Preventive Services Task Force (USPSTF) now recommends annual lung cancer screening (LCS) with LDCT for patients who are age 55-80, are either current smokers or former smokers who quit ≤ 15 years prior, and who have a ≥ 30 pack-year smoking history.[2] However, the literature of outcomes from and adherence to screening in usual clinical settings is limited. Both these factors may affect the risks, benefits and effectiveness of screening. The overall goal of this study is to examine the patient population and outcomes of those undergoing screening at a diverse tertiary care medical center with wide community referral catchment area, examine issues of adherence and patient-related factors associated with non-adherence, and build an ongoing cohort for LCS research.

1.2. Relevant Literature and Data

Previous studies of lung cancer screening are limited, given the novelty of the preventive health service. The largest population to undergo screening is the NLST cohort; however, this study population is significantly healthier, contained fewer active smokers, and had higher adherence (95%) due to the trial setting than patients eligible and undergoing screening in the United States.[1,3,4] The Veterans Affairs (VA) lung cancer screening demonstration project is the largest study of screening in a real-world setting. This study included 8 participating VA medical centers and two years of screening data, and demonstrated vastly different outcomes from NLST, including a higher prevalence of false-positive findings than would be expected.[5] However, this study may have limited validity outside of the VA setting as the majority of patients were men and many had a higher burden of comorbid illness.

Presently, the implementation of screening is suboptimal, and screening rates remain low.[6] Several studies, including our own prior work, have focused on assessing patient-, provider- and system-level factors to understand (and ultimately improve), utilization and referral.[5,7-11] The majority of these studies have found that the current state of patient and provider knowledge is likely limiting referral of eligible patients, and that there are key barriers and facilitators that can be addressed to improve access and ease of referral. On the other hand, there are few studies of adherence in patients referred to screening. Given both the high prevalence of abnormal screening studies and the requirement for a minimum of annual screening, high adherence is likely a necessary component to achieve benefits from screening. Adherence may be particularly important for effective smoking cessation coupled with screening, as smoking cessation rates may improve with repeated reinforcement.[12]

Further evaluation of screening would benefit from an assessment of screening participants, outcomes and key factors related to adherence in representative clinical settings.[13] We currently have 716 patients enrolled and tracked within our screening program, and the program is growing and expanding to multiple sites across the UW/SCCA system. This project also represents an opportunity to build a research database of our patients, and hopefully an

FHCRC IRB Approval
DEC 13 2018
Document Released Date

attractive database for future collaboration across systems to build a multicenter prospective cohort.

1.3. Compliance Statement

This study will be conducted with Fred Hutchinson Cancer Research Center (FHCRC) policies and procedures and all applicable Federal and state laws and regulations including 45 CFR 46 and the HIPPA Privacy Rule. Any episode of noncompliance will be documented. Collecting, recording, and reporting of data will be accurate and will ensure the privacy, health and welfare of research subjects during and after the study.

2. Study Objectives

2.1. Aims

Aim 1. Examine the demographic features of patients and referring clinics for lung cancer screening throughout the UW system.

Aim 2. Examine key outcomes from lung cancer screening in the UW system compared to outcomes from the NLST and the VA demonstration project.

Aim 3. Examine follow-up after initial screening. Compare adherence to follow-up during period before and after tracking, by Lung-RADS category, by smoking status and by patient demographic features.

Aim 4. Develop an ongoing research database of patients undergoing LCS within the UW system for future investigation.

2.2. General Schema of Study Design

The study will include: 1) a retrospective cohort study of electronic medical records for patients who have undergone LCS within the UW system (Aims 1-3); and 2) development of an ongoing prospectively enrolling cohort of subjects undergoing LCS and follow-up from LCS within the UW system.

2.3. Study Duration, Enrollment and Number of Sites

2.3.1. Date Range of Study

Subjects will be patients who underwent LCS at any UW site from 2012-present (duration of LCS program). These patients are currently identified in the SCCA Primordial tracking software. Ongoing enrollment will occur via review of the Primordial tracking software for new examinations every 3 months.

2.3.2. Total Number of Study Sites/Total Number of Subjects Projected

The study will be conducted at the FHCRC but will include patients undergoing screening throughout the UW system. Patients are referred for screening through a variety of providers including but not limited to primary care providers and pulmonologists. They undergo CT screening at 4 sites within the system: 2 UW Neighborhood clinic (UWNC) sites, the Seattle Cancer Care Alliance (SCCA) and the UW Medical Center (UWMC) with plans to expand to

Harborview Medical Center (HMC) within the year. 716 patients have had at least one LCS exam to date. The number of patients undergoing screening continues to increase with an estimated 240 new patients in 2017 alone. We estimate continued growth of the program as we prospectively enroll patients undergoing screening in coming years.

2.4. Study Population

2.4.1. Inclusion Criteria

Any patient undergoing a LDCT for the purposes of LCS at a UW system site between 01/2012 and present. Ongoing enrollment of patients undergoing LCS LDCT at any site. The patients are identified in the Primordial tracking database.

2.4.2. Exclusion Criteria

-Patient did not undergo any LDCT but are listed in Primordial
-Patient not enrolled into the Primordial tracking software
-Patients determined to have undergone a CT for reasons other than "lung cancer screening"

Of note, LDCT does not have other clinical uses at present, so unlikely that patients would undergo LDCT for reasons other than screening. Patients who underwent screening but were not eligible by standard criteria will still be included.

3. Study Procedures

3.1. Data Sources

Potential subjects will be identified through the Primordial tracking software. Patients who undergo LDCT lung cancer screening are uploaded into the software, with data input by Kaylee Peterson, ARNP, who works within the SCCA pulmonary section. Primordial provides a clinical tracking system for each exam and follow-up exam associated with lung cancer screening.

3.2. Data Elements to be Abstracted

Once subjects are identified, there will be two main sources of data, the Primordial software and the patients UW EHR (EPIC and ORCA).

The following elements will be abstracted through the Primordial dataset:
-MRN
-Date of Birth
-zip code
-insurance status
-race/ethnicity
-Screening exam date(s)
-Lung-RADS category
-incidental findings present
-Smoking history (dates, pack-years)
-Lung cancer diagnosis
-Lung cancer stage, histology

The following elements will be abstracted/confirmed through the EHR:

-insurance status
-race/ethnicity
-height
-weight
-review of screening CT report for:
    -nodules >4mm
    -confirmation of Lung-RADS
    -incidental findings
-follow-up images
-procedures related to lung cancer screening (bronchoscopy, EBUS, CT-guided biopsy, surgical biopsy/resection)
-results for patients undergoing screening
-last visit date in UW system
-referring provider type
-EHR based smoking data

4. Analysis

4.1. Statistical analysis

Data will be maintained in RedCap (as below) and spreadsheets will be imported into Stata (StataCorp, College Station, TX) for analysis. Standard descriptive summaries (e.g. means with standard deviations or medians with IQR for continuous variables such as age and percentage for categorical variables such as gender) will be used to summarize demographic variables, outcomes and adherence rates.

For adherence rate, subjects will be compared by demographic features, smoking status and Lung-RADS categories and be stratified by period before and after tracking (September 1, 2016) using Chi-squared or Fisher's exact test. If key factors of adherence are identified, a multivariable logistic regression model will be created.

5. Study Administration

5.1. Data Collection and Management

RedCap software (UW ITHS) will serve as the statistical software package for data abstraction from both Primordial and the EHR. Redcap is password protected with limited access controlled by PI. Redcap can convert output into XLS, STATA and other formats for analysis. The databases will be maintained on FHCRC servers (Pul_CC J-drive). Only members of the research study team will have access to the data.

After data abstraction, the medical record number will be converted to a unique study number and birthdate will be used to calculate age at exam. These will replace the medical record number in the dataset. A master data sheet will maintain the MRN and birthdate but will be maintained on the secure server.

5.2. Confidentiality

All data and records generated during the study will be kept confidential in accordance with FHCRC policies and HIPAA subject privacy. The investigator and other study team members will not use such data and records for any purpose other than conducting the study.

5.3. Regulatory and Ethical Considerations

5.3.1. Risk Assessment

This study poses minimal risks to the subjects. The main risks are breach of privacy and confidentiality. This study involves review of: tracking data which is collected for clinical and quality purposes, and medical records data collected for clinical purposes. Data will be kept on password-protected RedCap, and spreadsheets maintained in password-protected server on password-protected computer. Only a single linkage spreadsheet containing MRN and birthdate will be maintained in a separate location.

5.3.2. Potential Benefits of Study Participations

The main benefit to study participants will be in the form of quality improvement in the UW lung cancer screening program. Information from this study will inform future efforts to improve tracking and adherence which is maintained through Primordial software. A thorough summary of the screening program will also be useful to inform patients about local outcomes from screening. As subjects continue through annual screening, there may be benefits to subjects enrolled in the study.

5.3.3. Risk-Benefit Assessment

The potential benefits to the screening program, the subjects and future patients being screening both within and outside of the UW outweighs the minimal risk.

5.4. Informed Consent and HIPAA Authorization

5.4.1. Waiver of Consent

We are requesting a waiver of consent based on the following qualifications. For the **retrospective** portion of the study (all patient exams prior to IRB approval data): the research is limited to existing data; the data involves no more than minimal risk to the subjects; waiver of consent will not adversely affect the rights and welfare of the subjects, and the research could not be practicably carried out without the waiver.

For the **prospective** portion of the study, we are also requesting a waiver of consent based on the following qualifications: the research remains limited to data collected for clinical purposes with no additional data collection; the data involves no more than minimal risk to the subject; waiver of consent will not adversely affect the rights and welfare of the subjects. Despite prospective nature, the research is not practicable without the waiver: subjects are referred from screening from numerous clinics across to UW system and undergo CT screening at 4 (soon to be 5) distinct sites. As such, study personnel have no interaction with subjects. Moreover, any loss of subjects to non-consent would bias the study as universal enrollment of all screened subjects is necessary to study epidemiologic features (such as disparities in follow-up) of screening.

5.4.2. Waiver of HIPAA Authorization

We are requesting a waiver of HIPAA authorization based on the following qualifications: the use or disclosure of PHI involves no more than minimal risk, the research could not practicably

be conducted without the waiver or alteration, and the research could not be practicably conducted without access to and use of PHI.

The following procedures are in place to protect the identifiers from improper use and disclosure and destroy the identifiers at the earliest opportunity consistent with the conduct of the research.

During data abstraction, a unique study ID will be created for each patient. Only a single masterfile, maintained on the server and accessible by the PI, will link the study ID to the medical record. After completion of data collection, files used for analysis and accessible by the study team will not contain the medical record number, and birthdate will be converted to age. The protected health information will not be reused or disclosed to any other person or entity, except as required by law or for authorized oversight of the research project.

6. Safety Management

6.1. Clinical Adverse Events

Unanticipated problems involving risks to subjects and others will be monitored throughout the study

6.2. Adverse Events Reporting

No significant adverse events are expected as the study procedures are not greater than minimal risk. If any unanticipated problems related to the research involving risks to subjects or others happen during the course of the study, these will be reported to the IRB.

7. Publication

The results from this investigation will be presented at national conferences and published in peer-reviewed medical journals.

8. References

1. National Lung Screening Trial Research T, Aberle DR, Adams AM, et al. Reduced lung-cancer mortality with low-dose computed tomographic screening. The New England journal of medicine 2011;365:395-409.
2. Moyer VA, Force USPST. Screening for lung cancer: U.S. Preventive Services Task Force recommendation statement. Annals of Internal Medicine 2014;160:330-8.
3. Howard DH, Richards TB, Bach PB, Kegler MC, Berg CJ. Comorbidities, smoking status, and life expectancy among individuals eligible for lung cancer screening. Cancer 2015;121:4341-7.
4. Aberle Dr Fau - Adams AM, Adams Am Fau - Berg CD, Berg Cd Fau - Clapp JD, et al. Baseline characteristics of participants in the randomized national lung screening trial.
5. Kinsinger LS, Anderson C, Kim J, et al. Implementation of Lung Cancer Screening in the Veterans Health Administration. JAMA internal medicine 2017.
6. Jemal A, Fedewa SA. Lung Cancer Screening With Low-Dose Computed Tomography in the United States-2010 to 2015. JAMA oncology 2017.
7. Gesthalter YB, Koppelman E, Bolton R, et al. Evaluations of Implementation at Early-Adopting Lung Cancer Screening Programs: Lessons Learned. Chest 2017;152:70-80.

8. Iaccarino JM, Clark J, Bolton R, et al. A National Survey of Pulmonologists' Views on Low-Dose Computed Tomography Screening for Lung Cancer. Annals of the American Thoracic Society 2015;12:1667-75.
9. Crothers KA-Ohoo, Kross EK, Reisch LM, et al. Patients' Attitudes Regarding Lung Cancer Screening and Decision Aids. A Survey and Focus Group Study.
10. Triplette M, Kross EK, Mann BA, et al. An Assessment of Primary Care and Pulmonary Provider Perspectives on Lung Cancer Screening. LID - 10.1513/AnnalsATS.201705-392OC [doi].
11. Kanodra NM, Pope C, Halbert CH, Silvestri GA, Rice LJ, Tanner NT. Primary Care Provider and Patient Perspectives on Lung Cancer Screening. A Qualitative Study. Annals of the American Thoracic Society 2016;13:1977-82.
12. Kottke TE, Battista Rn Fau - DeFriese GH, DeFriese Gh Fau - Brekke ML, Brekke ML. Attributes of successful smoking cessation interventions in medical practice. A meta-analysis of 39 controlled trials.
13. Gould MK, Sakoda LC, Ritzwoller DP, et al. Monitoring Lung Cancer Screening Utilization and Outcomes in Four Cancer Research Network Sites. LID - 10.1513/AnnalsATS.201703-237OC [doi].
