# Supplementary material for: An alternating-intervention pilot trial on the impact of an informational handout on patient-reported outcomes and follow-up after lung cancer screening
Source: PLoS One. 2024 Apr 10;19(4):e0300352. doi: 10.1371/journal.pone.0300352 (PMC11006146; doi:10.1371/journal.pone.0300352)
Supplement: S3 File — (DOCX) [file pone.0300352.s003.docx]

**Supporting Information File 3.** Tables comparing PLCOm2012 risk scores between trial groups.

**Supplemental Table.** Comparisons of the PLCOm2012 lung cancer 6-year risk score between groups.

|  | **Sample Size** | | |  | **PLCOm2012**  **6-year LC risk (%)** | |
| --- | --- | --- | --- | --- | --- | --- |
|  | **Total** | **Missing risk score** | **With risk score** |  | **Median [IQR]** | **P-value*** |
| Group #1 |  |  |  |  |  | 0.99 |
| CAQ | 130 | 36 (28%) | 94 (72%) |  | 4.1 [2.4, 7.7] |  |
| Control | 100 | 20 (20%) | 80 (80%) |  | 4.3 [2.6, 6.7] |  |
| Group #2 |  |  |  |  |  | 0.94 |
| CAQ read | 44 | 12 (27%) | 32 (73%) |  | 4.1 [2.8, 6.5] |  |
| CAQ unread | 86 | 24 (28%) | 62 (72%) |  | 4.1 [2.3, 7.8] |  |

*Wilcoxon rank-sum or Kruskal-Wallis test comparing the lung cancer risk score between groups.

**Supplemental Table.** Missing risk factors used by the PLCOm2012 risk score* in the trial cohort (N = 130).

|  | **Missing** | |
| --- | --- | --- |
| **Variable** | **No.** | **%** |
| COPD | 40 | 17% |
| Family history | 34 | 15% |
| BMI | 10 | 4% |
| Race/ethnicity | 6 | 3% |
| Education | 2 | 1% |
| Cigarettes/day | 1 | <1% |

*Based on variables available in the CAQ survey and lung cancer screening repository.

**Supplemental Table.** Missing risk factors used by the PLCOm2012 risk score* (besides education) in the responder vs. non-responder cohort (N = 389).

|  | **Missing** | |
| --- | --- | --- |
| **Variable** | **No.** | **%** |
| COPD | 93 | 24% |
| Race/ethnicity | 77 | 20% |
| Family history | 71 | 18% |
| Cigarettes/day | 32 | 8% |
| Duration of smoking | 32 | 8% |
| BMI | 21 | 5% |
| Duration of quitting | 10 | 3% |
| Current smoker | 3 | 1% |

*Based on variables available in COALS only.
